# High-Density “Windowpane” Coordination Patterns of Water Clusters and Their NBO/NRT Characterization

**DOI:** 10.3390/molecules27134218

**Published:** 2022-06-30

**Authors:** Frank Weinhold

**Affiliations:** Theoretical Chemistry Institute and Department of Chemistry, University of Wisconsin-Madison, Madison, WI 53706, USA; weinhold@chem.wisc.edu

**Keywords:** supramolecular chemistry, hydrogen bonding, water clusters, natural bond orbitals, natural resonance theory, natural bond orders, Grotthuss proton ordering, water wires, glassy water, quantum cluster equilibrium

## Abstract

Cluster mixture models for liquid water at higher pressures suggest the need for water clusters of higher coordination and density than those commonly based on tetrahedral H-bonding motifs. We show here how proton-ordered water clusters of increased coordination and density can assemble from a starting cyclic tetramer or twisted bicyclic (Möbius-like) heptamer to form extended *Aufbau* sequences of stable two-, three-, and four-coordinate “windowpane” motifs. Such windowpane clusters exhibit sharply reduced (~90°) bond angles that differ appreciably from the tetrahedral angles of idealized crystalline ice I_h_. Computed free energy and natural resonance theory (NRT) bond orders provide quantitative descriptors for the relative stabilities of clusters and strengths of individual coordinative linkages. The unity and consistency of NRT description is demonstrated to extend from familiar supra-integer bonds of the molecular regime to the near-zero bond orders of the weakest linkages in the present H-bond clusters. Our results serve to confirm that H-bonding exemplifies resonance–covalent (fractional) bonding in the sub-integer range and to further discount the dichotomous conceptions of “electrostatics” for intermolecular bonding vs. “covalency” for intramolecular bonding that still pervade much of freshman-level pedagogy and force-field methodology.

## 1. Introduction

The earliest applications of ab initio natural bond orbital (NBO) analysis [1,2,3,4] consistently revealed a “donor–acceptor” (resonance–covalency-type “charge transfer”) picture of hydrogen bonding that was sharply at odds with then-prevalent “electrostatic” conceptions of intermolecular interactions [5,6]. Although the IUPAC *Gold Book* definition of H-bonding was subsequently revised to acknowledge the importance of covalency in H-bonding [7], superficial “dipole–dipole” rationalizations of H bonding continue to survive in many freshman-level expositions [8]. Arguments against the charge-transfer picture or in support of classical-type long-range, multipole, or “electrostatically driven” conceptions of H-bonding continue to appear [9,10] (vs. replies in [11,12,13]) in the research literature, and similar simplifying approximations persist in the empirical force fields of popular molecular dynamics (MD) simulation methods [14] that are commonly adopted to describe H-bonding in condensed phases.

The daunting task of describing macroscopic phases of liquid water or other H-bonded fluids may seem to demand the drastic long-range approximations of intermolecular (“noncovalent”) interactions as compared to the exchange-type (“covalent”) interactions of the short-range molecular regime. However, a more practical and accurate approach to describing intermolecular H-bonding is achieved by adopting *supramolecular clusters* [15] {*C*_n_} as the conceptual “building blocks” of the macroscopic liquid-phase description, based on the known *continuity* of high-density liquid and low-density gaseous phases around the fluid critical point [16]. More specifically, quantum cluster equilibrium (QCE) theory [17,18,19] provides a practical numerical implementation of such “cluster mixture” [20,21,22,23,24,25] modeling of macroscopic phase properties, based on accurate values of electronic and vibrational properties of H-bonded {*C*_n_} clusters that can be obtained at any chosen ab initio or density functional theory (DFT) level. The key input for QCE-based thermodynamic modeling of an aqueous phase is the data set of supramolecular clusters whose self-consistent (*T*,*P*)-dependent equilibrium populations are determined from the computed partition functions for each cluster by the standard methods of quantum statistical thermodynamics [26].

Among the many H-bonded fluids of practical interest, water itself presents the most studied yet still most perplexing phase behavior of the terrestrial regime [27]. Even the microscopic structure and properties of “ordinary” liquid water under near-ambient conditions remain matters of controversy [28]. Further mysteries surround the phase behavior of water at higher temperatures and pressures, where both theory [29,30,31,32] and experiments [33,34,35,36] have suggested the existence of an alternative high-density phase of liquid water that could lead to a liquid–liquid critical point and an exotic new domain of thermodynamic behavior near 220 K and 1–2 kbar.

**Figure 1:**
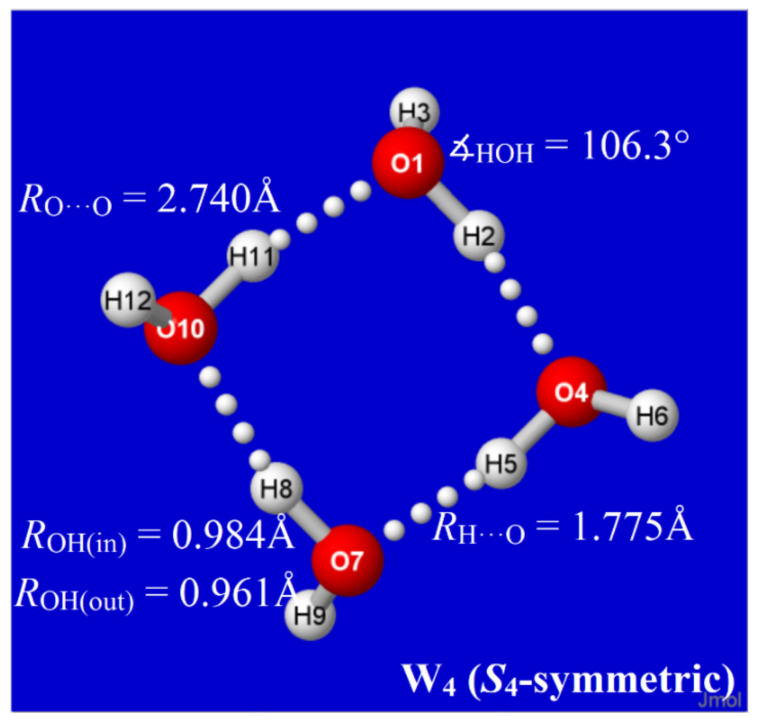
Equilibrium structural properties of cyclic (H_2_O)_4_ “windowpane” cluster (B3LYP/6-311++G** level).

The primary goal of present work was to computationally search for a new class of water clusters {*W*_n_} based on the quadrilateral (“windowpane”) coordination motif of the cyclic tetramer (Figure 1) that might contribute to equilibrium QCE populations in the neighborhood of the proposed high-density phase. In each case, we restricted attention to clusters that maintain maximal Grotthuss-type proton ordering for the powerful effects of cooperative stabilization [37,38], as exemplified by the clockwise ordering of in-ring OH bonds in the view of Figure 1. The near −90° coordination angles of the windowpane class correspond to reduced next-neighbor distances and increased mass/volume ratios compared to the characteristic tetrahedral angles and chair–hexagon coordination motifs of ice-I-like clusters. The search for cooperatively stabilized windowpane clusters is organized in *Aufbau* fashion toward increasing numbers of fully four-coordinate sites that more adequately sample the intermolecular interactions expected to dominate in the phase behavior of the low-temperature and high-pressure regime. The resulting windowpane clusters can serve as computational input for subsequent QCE studies to examine their possible role in the equilibrium cluster distributions of the water-phase diagram.

A secondary goal of this study was to characterize each computed cluster in deeper conceptual terms that can clarify distinctive features of the underlying H-bond interactions. Such characterization should include aspects of overall cluster stability, strengths of individual coordinative linkages, shifts in atomic charge distribution, and other orbital-level features of free vs. coordinated water molecules. For these purposes, we employed NBO analysis [39,40] to obtain localized descriptors of molecular and intermolecular bonding features. Of particular interest are natural resonance theory (NRT) bond orders [41], which are expected to exhibit useful correlations with bond lengths [42,43], bond energies [44,45], bond stretching frequencies [46,47,48], NMR ^1^J and ^1h^J spin-coupling constants [49], and other experimentally measurable properties.

Although the present study of novel water clusters was primarily directed toward equilibrium thermodynamic properties, it is important to note that such studies can also yield information on the kinetics and mechanisms of water cluster reactions. This is particularly true when, as in the present case, each cluster of the class is created in a sequential *Aufbau* manner from a previous member, e.g., by successive dimer additions of the form

*W*_k_ + *W*_2_ ⇌ *W*_k+2_ (1)

where *W*_k_ = (H_2_O)_k_ is a *k*-mer of a chosen coordination pattern. Analogous to elementary A + B ⇌ C chemical reactions, one can compute the transition state (*W*_k_···*W*_2_)^‡^ and other features of the intrinsic reaction coordinate [50] (IRC) for each such cluster reaction. Similarly, for other cluster species satisfying the simultaneous QCE equilibrium conditions,

*W*_j_ + *W*_k_ ⇌ *W*_j′_ + *W*_k′_ (j *+* k *=* j′ + k′) (2)

standard quantum chemical methods can be employed to determine transition-state features and associated absolute rate constants along the associated IRC [51]. However, such deeper mechanistic aspects of cluster formation were not addressed in the present work.

## 2. Computational Methods

For direct comparisons with many previous chemical applications in the NBO/NRT literature [52,53], we employed the familiar B3LYP/6-311++G** level of hybrid density functional theory for all geometry optimizations and energy evaluations of the present work. As shown elsewhere [54,55], realistic treatment of thermodynamic properties requires balanced treatment of energetic (primarily electronic) and entropic (primarily vibrational) contributions to free energy. All species were fully optimized and checked for vibrational stability with standard options of the *Gaussian-16* program [56]. NBO/NRT analyses were completed with the *NBO7* program [57,58] in interactive *G16/NBO7* configuration. Structural and orbital graphics were obtained with the *NBOPro7@Jmol* utility program [59]. For NRT analyses of larger clusters, keyword selections for enlarged dynamic memory and the number of resonance structures were required to obtain fully converged bond orders. Ready-to-run input files containing optimized cartesian coordinates and keyword input for each cluster are included in the Supporting Information (SI). As shown particularly in ref. [54], many DFT variants and additional “corrections” (for dispersion, counterpoise, etc.) give qualitatively similar results for individual cluster structures and relative energies, even if some choices prove “best” for a particular thermodynamic comparison. The provided SI files allow re-optimization of cluster structures for alternative method/basis levels of choice. 

## 3. Sequential Aufbau of 2-, 3-, 4-Coordinate Windowpane Water Clusters

The properties of each water cluster *W*_k_ of an envisioned class are dictated by its specific H-bond coordination pattern. As primary descriptors of this pattern, we expect that each water molecule may generally be involved in two-, three-, or four-coordinate H-bonding to other molecules of the cluster (with singly coordinated “dangling” molecules excluded in leading clusters of the equilibrium thermodynamic distribution). For labeling purposes, the coordination pattern of each cluster may be usefully described by the number of quadruply (*q*), triply (*t*), or doubly (*d*) coordinated sites, appended as pre-superscripts (viz., ^q,t,d^W_n_) to the cluster symbol. In this notation, the cyclic water tetramer of Figure 1 is labeled ^0,0,4^W_4_, with each monomer doubly coordinated in chain-like linkages to the substrate.

**Figure 2:**
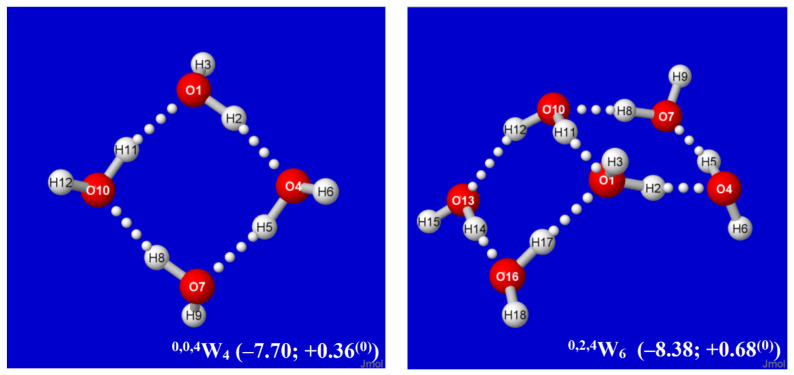

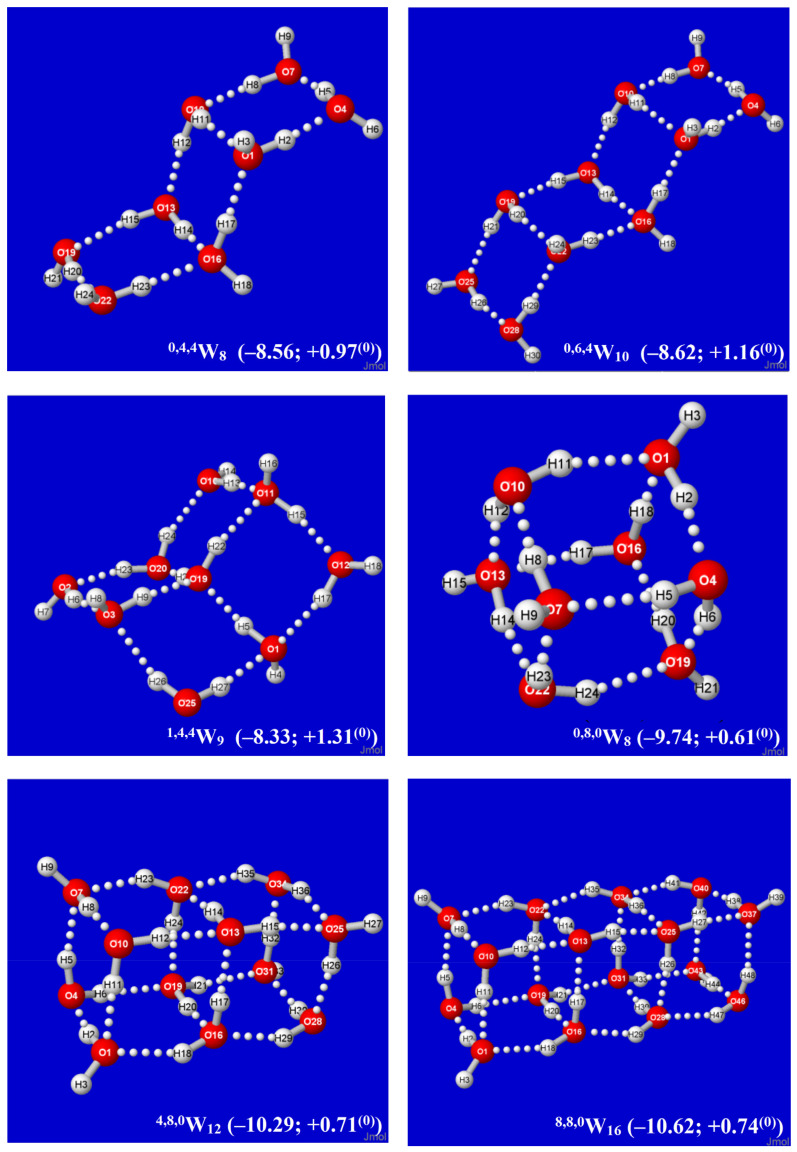
Calculated *Aufbau* sequence of windowpane clusters ^q,t,d^W_k_ from starting cyclic tetramer ^0,0,4^W_4_ (**upper left**), showing parenthesized per-monomer changes (kcal/mol) in energy (Δ*E*) and Gibbs free energy (Δ*G*^(0)^) from free water molecules in each panel.

The structural logic for sequential *Aufbau* construction of windowpane clusters is straightforward. Starting from an existing cluster of this class, such as the cyclic water tetramer of Figure 1, one can choose any edge-type coordination (such as that between O(1) and O(10) in Figure 1) as a “base” for a new windowpane by attaching a water dimer in parallel fashion with two new H-bonds, as shown in the left panel of Figure 2. For maximum stabilization in forming this new H-bond attachment (e.g., from emanating H(12) at O(10)), the Grotthuss-type proton ordering should be continued around the edges of the newly formed windowpane that joins to O(1). The net result of this particular attachment is that sites O(10) and O(1) become tri-coordinate (*t* → *t* + 2), while other sites remain di-coordinate, leading to an overall ^0,0,4^W_4_ → ^0,2,4^W_6_ change in labeling. Some of these clusters, such as ^0,0,4^W_4_ itself or the cubane-like ^0,8,0^W_8_ described below, are featured in many previous cluster investigations, but the emphasis here is on hierarchical families of clusters that can be associated with a well-defined mechanistic *Aufbau* sequence of dimer additions, particularly leading to higher four-coordinate (*q*-type) motifs.

By alternating the sign of folding angles between panes, such additions can be continued indefinitely in “ladder-like” procession, as shown in successive panels of Figure 2. Each panel of Figure 2 includes (in parentheses) the per-monomer energy and standard-state Gibbs free energy change with respect to free water molecules, which serve to exhibit the important cooperative (nonadditive) effects of Grotthuss-ordered coordination patterns. The first four panels (^0,0,4^W_4_, ^0,2,4^W_6_, ^0,4,4^W_8_, ^0,6,4^W_10_) show the addition of successive rungs to the ladder pattern, up to the four-pane member. The ensuing ^1,4,4^W_9_ (row 3, left) is the alternative “2 × 2” four-pane cluster, which adopts a buckled saddle-shape deformation from planarity with a central four-coordinate monomer. From the starting two-pane ladder (^0,2,4^W_6_) at the upper right, one can also attempt to add another rung that curls backward (*E*-like) rather than forward (*Z*-like), but this optimizes to the cubane-like ^0,8,0^*W*_8_ cluster (row 3, right). The cubane motif becomes an evident building block for extensions to two-cube (^4,8,0^*W*_12_), three-cube (^8,8,0^*W*_16_), or longer rod-like clusters, as illustrated in the final row of the figure. 

**Figure 3:**
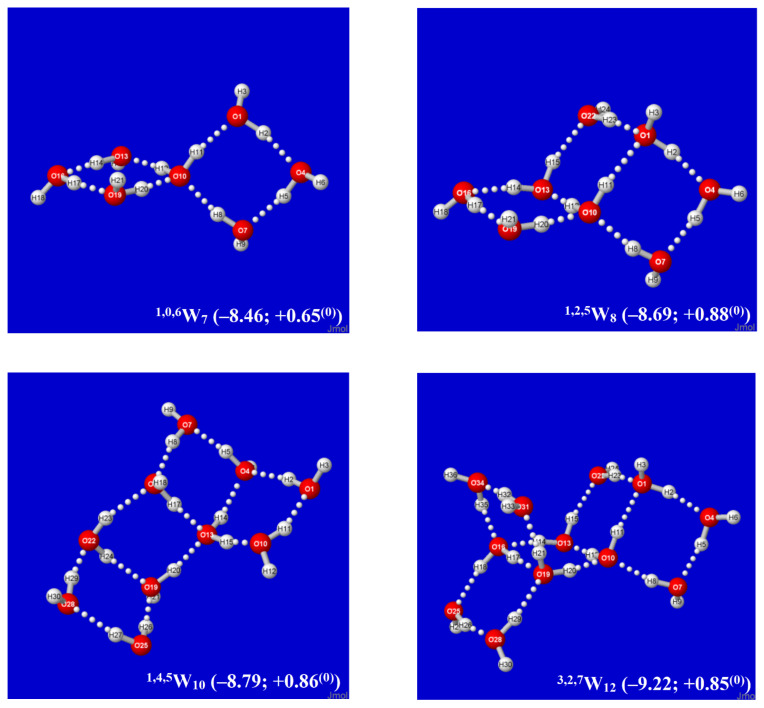

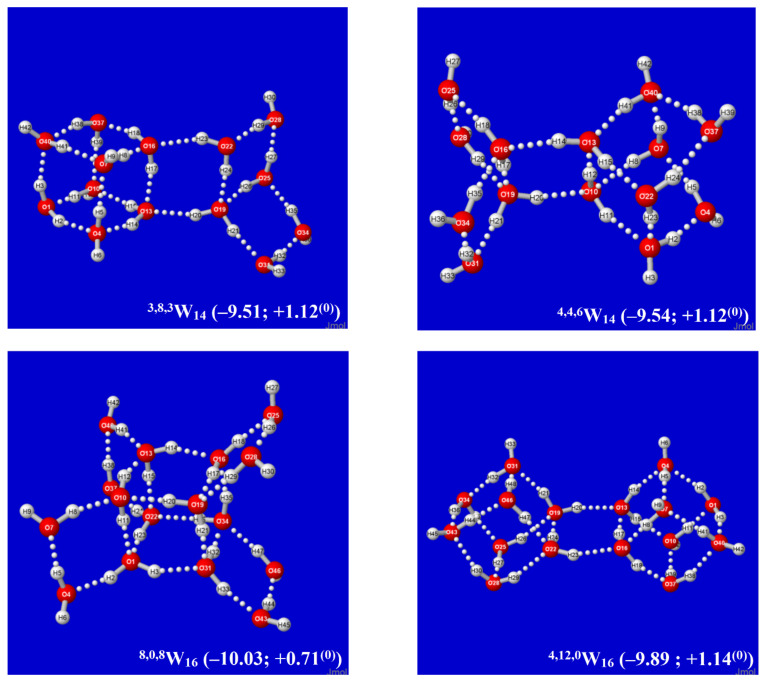
Similar to Figure 2, for successive ^q,t,d^W_k_ windowpane clusters built from the Möbius-like ^1,0,6^W_7_ cluster (**upper left**).

An alternative *Aufbau* starting point is provided by the twisted two-pane (^1,0,6^W_7_) cluster shown in the upper-left panel of Figure 3. This cluster features “Möbius-like” coordination with a continuous Grotthuss-ordered chain passing twice through the unique four-coordinate central monomer to form a closed loop. Remaining panels of Figure 3 show selected clusters that are obtained by successive Grotthuss-ordered dimer additions to ^1,0,6^W_7_, aimed at increasing *q* numbers of saturated four-coordinate sites. The resulting structures all incorporate the higher density coordination angles of the windowpane motif, but they exhibit irregular overall shapes that appear suitable as possible contributions to bulk liquid or amorphous solid phases. As seen in Figure 2 and Figure 3, the ^8,8,0^W_16_ cluster (Figure 2, lower right) achieves the largest number of three- and four-coordinate sites (*q* = *t* = 8) and the deepest per-monomer energy (−10.62 kcal/mol) in the depicted sequences. However, whether some or all of these clusters contribute significantly to known roots of the QCE equations, or whether (like the buckyball-type clathrate clusters previously studied [60]) they can serve as leading contributors to entirely new roots (phases) of the QCE phase diagram remains to be investigated.

**Figure 4:**
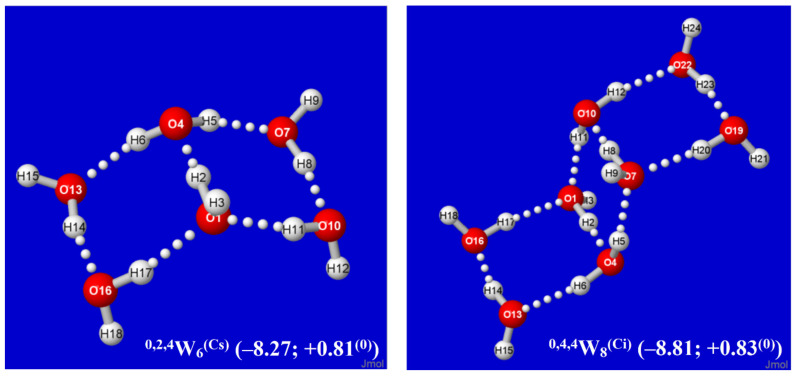
Alternative higher-symmetry ^0,2k,4^W_2k+4_^(sym)^ clusters (*k* = 1,2), one (*C*_s_) of higher energy, the other (*C*_i_) of lower energy than the corresponding low-symmetry structure of Figure 2.

It is evident that each *Aufbau* cluster shown in Figure 2 and Figure 3 may have alternative isomeric rearrangements of the proton network without altering the *q/t/d* descriptors of O···(H)···O coordination linkages. Such alternative ^q,t,d^W_n_^(alt)^ isomers may have higher point group symmetry, different proton orderings (e.g., Grotthuss cycles around individual panes rather than overall periphery), and higher or lower energy than the *Aufbau*-derived clusters described above. Figure 4 displays two such alternative high-symmetry forms of the ^0,2k,4^W_2k+4_^(sym)^ sequence (*k* = 1, 2), with respective *C*_s_ (*k* = 1), *C*_i_ (*k* = 2) symmetry. The *C_s_*-symmetric ^0,2,4^W_6_^(Cs)^ structure (Figure 4, left) is slightly higher in energy than ^0,2,4^W_6_ of Figure 2, but *C*_i_-symmetric ^0,4,4^W_8_^(Ci)^ (Figure 4, right) is slightly lower in energy than its low-symmetry counterpart in Figure 2. The inherent chirality of the coordination pattern about *each* O atom of higher-coordinated water clusters of Figure 2 and Figure 3 indicates that reduced symmetry (net chirality) is a high-probability feature of equilibrium water cluster distributions in any phase involving their participation.

Note that although H-bonds are considered weak noncovalent attractions, the cumulative energy release from larger cluster formation (viz., Δ*E* ≈ 170 kcal/mol for the ^8,8,0^W_16_ cluster) can readily exceed that necessary to dissociate a strong covalent bond, as in the ion pair clusters involved in self-dissociation (pH) of liquid water [54,55]. The per-monomer free energies of formation shown in Figure 2 and Figure 3 remain slightly positive under standard-state conditions, but the windowpane clusters are expected to gain increased stability relative to the ice-like clusters of the near-ambient regime as pressure increases. Full thermochemical and vibrational spectroscopic values for each cluster are included with the optimized coordinates in SI.

## 4. Natural Atomic Charge and Bond Order Characterizations

Among the many descriptors provided by NBO analysis, the natural atomic charges {*Q*_A_} and interatomic bond orders {*b*_AB_} are most intimately associated with traditional empirical concepts of chemical bonding theory. Long-held perceptions of *dichotomy* between intra- vs. intermolecular forces (viz., “covalency” for chemical bond formation (*b*_AB_ = 1, 2, 3,...) vs. “electrostatics” for H-bond formation (*b*_H···O_ ≈ 0.1–0.2)) have long impeded true progress in the supramolecular domain. Demonstrations of how quantal *Q*_A_, *b*_AB_ descriptors extend seamlessly across the supposed divide can therefore serve to refute the obsolete dipole–dipole conceptions of H-bonding (and other so-called “non-covalent” interactions) that still pervade freshman-level pedagogy and classical force-field methodology. In the present section, we wish to test the usefulness of NBO/NRT-based *Q*_A_, *b*_AB_ descriptors when applied to the large data base of windowpane water clusters as described above.

### 4.1. General Features of Donor–Acceptor Interactions in Water Clusters

**Figure 5:**
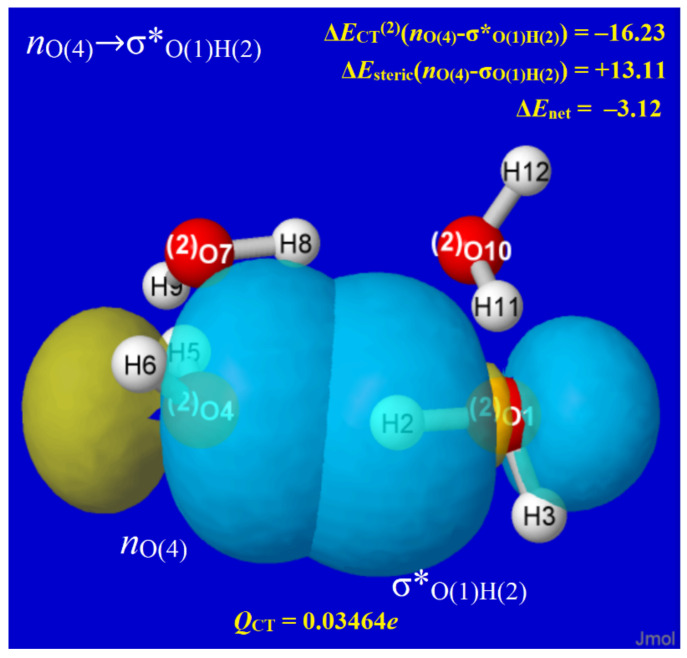
Pre-orthogonal (PNBO) depiction of *n*_O_→σ*_OH_ orbital interaction in one H-bond of W_4c_, with energetic (kcal/mol) and charge transfer (*e*) details as insets (see text).

In every H-bond of every water cluster, NBO analysis reveals the characteristic *n*_O_→σ*_OH_ donor–acceptor (“charge transfer”) interaction that transfers a slight electronic charge (*Q*_CT_) from the oxygen lone pair (*n*_O_) of the Lewis base (LB) site into the valence antibond (σ*_OH_) of the proximal Lewis acid (LA) site. Figure 5 depicts the *n*_O_-σ*_OH_ interaction for one of the H-bonds of W_4c_, showing the strongly overlapping forms of pre-orthogonal PNBOs deep inside van der Waals contact. The insets show details of the interaction that are routinely provided in NBO output, including (in kcal/mol; upper right) the second-order perturbative estimate of *n*_O_-σ*_OH_ donor–acceptor attraction (Δ*E*_CT_^(2)^), the corresponding steric opposition of *n*_O_-σ_OH_ donor–donor repulsion (Δ*E*_steric_), and the net binding energy (Δ*E*_net_). The known high transferability of NBOs [61] then assures that the individual *n*_O_, σ*_OH_ orbitals are quite similar to those in water monomer and dimer as well as other windowpane clusters. However, one can also recognize the slight misalignments of ring strain that lower PNBO overlaps throughout the windowpane series and lead to the nuances in charge distribution, structure, and bond strength discussed below.

**Figure 6:**
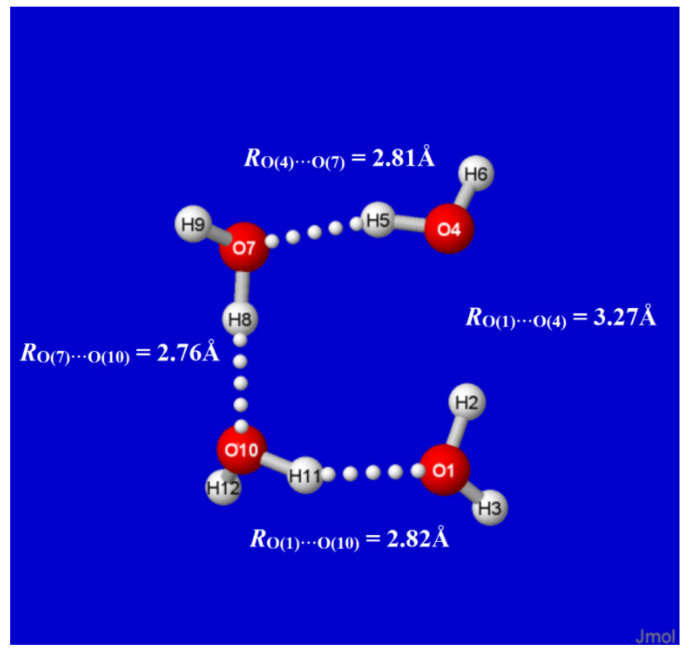
$DEL (partially)-reoptimized structure of original W_4c_ cluster (Figure 1), showing effects of deleting the single *n*_O(4)_→σ*_O(1)H(2)_ interaction of Figure 5 (at the point where the maximum number of optimization steps was completed).

Alternatively, the effects of *n*_O(4)_→σ*_O(1)H(2)_ interaction can be quantified by *deleting* this single specific matrix element from the DFT calculation (with standard $DEL keylist options [62]) and recalculating the energy and reoptimized geometry as though it were absent in nature. As shown in Figure 6, this single deletion “breaks” the O(4)···H(2)−O(1) hydrogen bond (and initial *S*_4_ symmetry) to give an open-chain structure with *R*_O(1)···O(4)_ separation increased by ~0.5 Å to near-van der Waals contact distance. The monomers at each chain terminus also reorient to near coplanarity (contrary to the ~120° dihedral twisting of the two remaining monomers), thereby allowing partial re-gain of *n*^(σ)^_O(4)_→σ*_O(1)H(2)_ attraction with the weaker *in*-plane *n*^(σ)^_O(4)_ lone pair of O(4). By such $DEL deletion searches, one verifies that the specific *n*_O(4)_→σ*_O(1)H(2)_ interaction is the unique “smoking gun” that is both *necessary and sufficient* for characteristic H-bonding between O(1) and O(4) monomers.

All such NBO-based energetic and $DEL deletion descriptors can be obtained for other windowpane clusters of Figure 2 and Figure 3. In the following, we focus instead on subtleties of the charge distributions and H-bond strengths that relate to the interesting cooperative effects of the highly ordered proton patterns (“water wires”) formed by the H-bond networks.

### 4.2. Natural Atomic Charge Distributions

**Figure 7:**
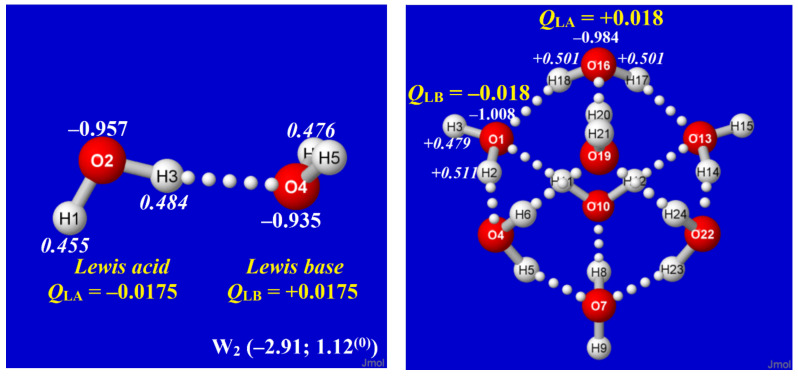
Natural atomic charges for H (italics; white) and O (plain text; white) atoms of water dimer (**left**) and cubane-like ^0,8,0^W_8_ cluster (**right**), with corresponding net charges (yellow) of formal Lewis acid (*e*-acceptor) and Lewis base (*e*-donor) water molecules in each species, showing the *reversal* of apparent charge flow in the two cases. (Parenthesized per-monomer energy and free energy for W_2_ also allow direct stability comparisons with clusters of Figure 2, Figure 3 and Figure 4).

In principle, the simple water dimer (W_2_) might be seen as the fundamental conceptual building block for studies of electronic charge distribution and stability in clusters of higher complexity. However, Figure 7 exhibits the detailed comparisons of H atom (italic) and O atom (plain text) natural charges in W_2_ vs. cubane-like ^0,8,0^W_8_ to show the surprising *contrasts* between these species. In the two panels of Figure 7, the O(1) and O(16) monomers of the cubane cluster (right) are, respectively, the direct analogs of O(4) and O(2) monomers in the dimer (left), yet the net charges on the monomers of the dimer are directly *opposite* those in the cluster. Similar contrasts between charge distributions of the supposed “building block” dimer and those of higher coordination complexes are found throughout the clusters of Figure 2 and Figure 3.

How can the conflicting charge patterns of Figure 7 be rationalized? At the termini of each H-bond are two water monomers that can be identified as the LB (formal *e*-pair “donor”) and LA (formal σ*_OH_ “acceptor” vacancy). In the simple water dimer, the *n*_O_→ σ*_OH_ donor–acceptor interaction necessarily results in net charge transfer (ca. 0.017*e*) from LB to LA (Figure 7, left), resulting in the LB^δ+^···LA^δ−^ charge pattern. However, in more complex water clusters, the surroundings of any chosen H-bond may be seen as a network of “water wires” that allow charge to redistribute as necessary to optimize overall cluster stability. Specifically, the multiple network connections allow electronic charge to be redistributed to achieve near *neutrality* at *q*- or *d*-coordinated sites, whose equal numbers of donor and acceptor interactions can be tuned to avoid capacitive build-up. However, at *t*-coordinated sites, which necessarily have an imbalance of donor (*t*_d_, LB) vs. acceptor (*t*_a_, LA) connections, it becomes advantageous to confer excess *anionic* charge on *t*_d_ sites (increasing LB strength) and *cationic* charge on *t*_a_ sites (increasing LA strength), thus leading to the commonly observed LB^δ−^···LA^δ+^ charge pattern. 

**Figure 8:**
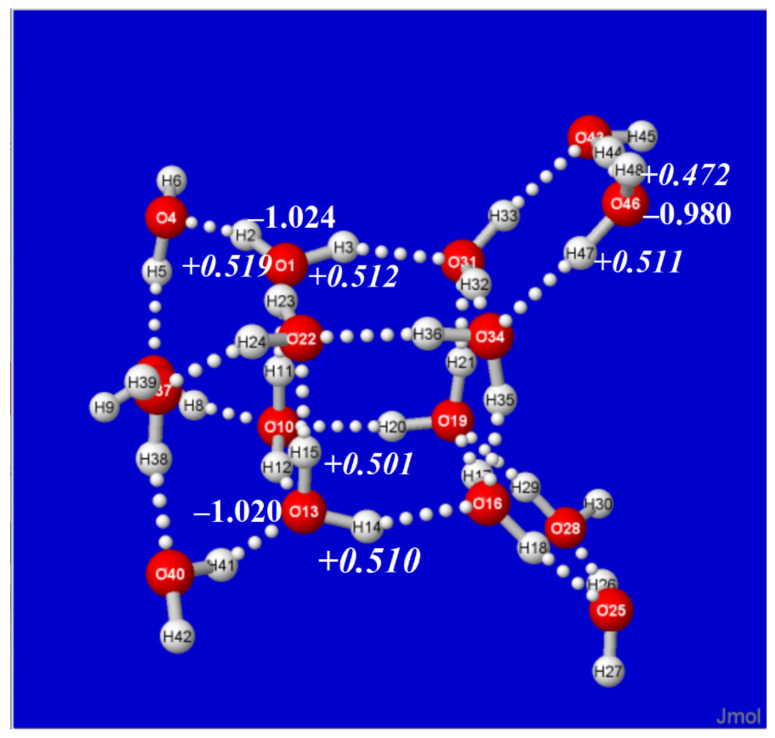
Similar to Figure 7, for representative quadruply (*q*-type) coordinated O(1)H(2)H(3) and O(13)H(14)H(15) molecules of the cubane-like core, and doubly (*d*-type) coordinated O(46)H(47)H(48) molecule on a bridged wing of the ^8,0,8^W_16_ cluster.

To illustrate these propensities of cluster charge distribution, Figure 8 displays selected *Q*_O_ (plain text) and *Q*_H_ (italic) atomic charges of the ^8,0,8^W_16_ cluster for two *q*-type sites (centered at O(1), O(13)) and one *d*-type site (at O(46)), showing the significantly reduced net monomer charges compared to those of the water dimer. More complete listings of monomer charge values and coordination type at each O atom for all clusters of this study are included in SI, as illustrated for the ^8,0,8^W_16_ cluster in Table 1. The subtle variations in molecular charge indicate the extreme “feedback” sensitivity to every detail of the surrounding H-bond network, showing that overall network topology has taken precedence over characteristics of the water dimer (single H-bond) “building block” of which the network is composed.

### 4.3. Natural Bond Order Correlations

The distended shapes of windowpane clusters provide clear evidence of the severe effects of “ring strain” in altering the network O−H···O bonds from the idealized geometries of isolated H-bonds in binary complexes. Nevertheless, one expects that network H-bonds should continue to exhibit the robust correlations with NBO/NRT measures of bond order and charge transfer that were previously demonstrated for free binary H-bonded species [63]. We now turn to examining the supramolecular extension of such correlations for the classical bond order–bond length (BOBL) relationships that have long been fruitfully employed in the integer (single-, double-, triple-, etc., bond) range of covalent bonding in molecules [42,43]. 

**Figure 9:**
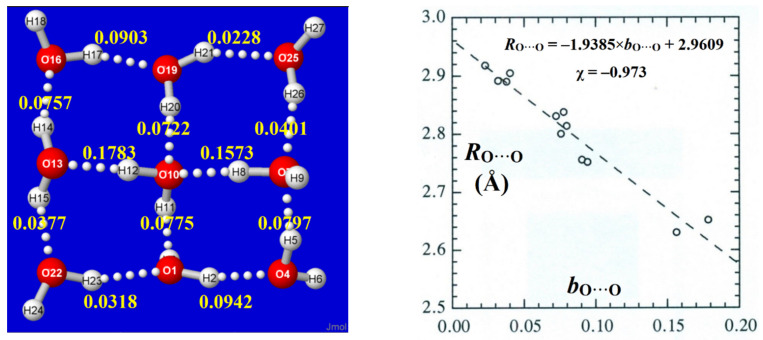
Calculated NRT bond orders *b*_O···O_ of the ^1,4,4^W_9_ windowpane cluster (**left panel**), showing their excellent BOBL correlation (Pearson χ = −0.973) with optimized *R*_O···O_ bond lengths (**right panel**).

A simple example of such BOBL correlations is illustrated in Figure 9 for the ^1,4,4^W_9_ windowpane cluster of Figure 2. For each O···H−O linkage, the total *b*_O···O_ bond order is obtained as the sum of *b*_O···H_ (major) and “long-bond” [64] *b*_O^O_ (minor) contributions,

*b*_O···O_ = *b*_O···H_ + *b*_O^O_ (3)

with sub-integer values ranging from 0.02 to 0.18 in this simple cluster. As shown in the right panel, the BOBL correlation is of excellent quality (Pearson correlation coefficient χ ≈ −0.97), and the least-squares regression line (shown in the inset) allows close prediction of *R*_O···O_ distances to near the 0.01Å level(!), despite the fact that NBO/NRT descriptors receive *no* input from real-space molecular geometry or spatial distribution of electron density. Thus, the resonance–covalency concepts underlying NRT bond order evaluations appear to extend seamlessly into this *sub*-integer range of weak H-bonding in clusters, practically as well as the familiar *supra*-integer range of strong covalent bonding and resonance in molecules. 

More complex three-dimensional structures of windowpane clusters obstruct clear visual representation of all relevant *b*_O···O_ bond orders and tend to show additional effects of ring strain. Comprehensive listings of *b*_O···O_ bond orders and *R*_O···O_ distances (Å) for all H-bonds in all clusters (keyed to the atom numberings of Figure 2 and Figure 3) are presented as tables in SI, as exemplified for the ^4,4,6^W_14_ cluster in Table 2. In this case, the *b*_O···O_-*R*_O···O_ correlation is found to be weaker, but still of reasonably high quality (χ ≈ −0.91), reflecting the heterogeneities of higher-coordination motifs.

**Figure 10:**
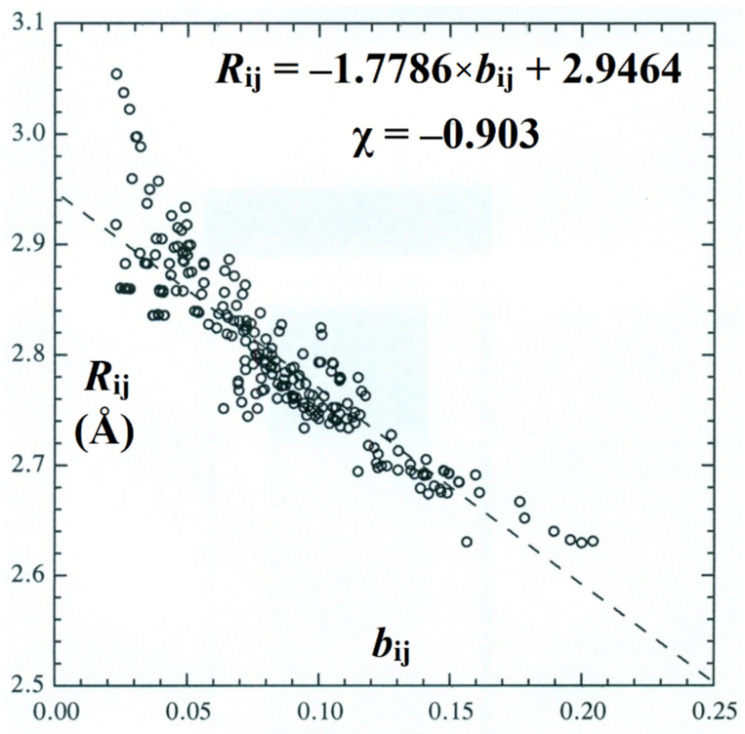
Scatter plot, least-squares regression line, and Pearson correlation coefficient (χ) for *b*_ij_-*R*_ij_ BOBL correlations of all (~250) H-bonds in the clusters of Figure 2 and Figure 3.

It is also of interest to examine the global BOBL correlations for *all* windowpane clusters of the present work, covering ca. 250 individual *b*_O···O_-*R*_O···O_ H-bonded pairs in a broad variety of coupled coordination motifs. Figure 10 displays the BOBL scatter plot, least-squares regression line, and Pearson correlation coefficient for this entire data set of hydrogen bonds, showing the strong correlation (χ = −0.90) that persists in spite of increasingly heterogeneous cluster topologies. 

The degraded accuracy of the linear least-squares regression fit in Figure 10 (compared, e.g., to that in Figure 9) can be primarily attributed to the upward deviations from linearity that are evident near *b*_ij_ → 0. However, it is important to recognize that these deviations are *required* on physical grounds, because intermonomer separation should asymptotically *diverge* (*R*_ij_ → ∞) as bond order vanishes (*b*_ij_ → 0). Indeed, only the higher-order connectivity of the H-bond network prevents such asymptotic dissociation when any single H-bond is severed, so the proper appearance of such nonlinearity in the *b*_ij_ → 0 limit serves to further confirm the resonance–covalent nature of H-bonding even in this range of interaction strengths near the limit of chemical interest. 

## 5. Conclusions

In the present work, we have employed standard density functional methods to computationally characterize a broad variety of unusual “windowpane” clusters that may play a role in the high-density fluid phase(s) of water. Despite their diverse topological forms and unusual angular features, we have demonstrated that these clusters are fully compliant with water’s known facility in forming doubly (*d*-type), triply (*t*-type), and quadruply (*q*-type) coordinative linkages to other water molecules, leading to multiply connected (“water-wired”) networks of increasing energetic stability when proper Grotthuss-type proton ordering is maintained. The *Aufbau* construction approach also suggests the mechanistic sequence by which such Grotthuss-ordered clusters can readily form from successive aggregation with water dimers.

We have also employed natural bond orbital (NBO) and natural resonance theory (NRT) analysis tools to demonstrate the consistency and accuracy with which H-bonding in these clusters conforms to the general conceptual picture of *resonance–covalency* (“charge transfer”) as the authentic origin of intermolecular O−H···O attractions. The charge flows and adaptive bond order and structural shifts in these clusters are shown to obey familiar bond order–bond length (BOBL) correlations with high accuracy (|χ| > 0.9). Moreover, the BOBL correlations also exhibit the expected *deviations* from linearity in the asymptotic limit of vanishing bond order where *R*_O···O_ distance becomes divergent. Although connections can be shown between NBO and Bader-type descriptors [65], we believe that the NRT bond orders of the present work provide broader predictive utility and more nuanced inclusion of resonance effects than the topological descriptors as employed in previous studies of water clustering (e.g., [66]).

The reader is reminded that “correlation is not causation.” Nevertheless, the *continuity* of robust BOBL correlations that stretch across the broad extremes of supramolecular (sub-integer) vs. molecular (multi-integer) bond orders strongly implies their *shared* origin in unified “covalency” concepts, contrary to the dichotomous viewpoint that still dominates freshman-level teaching of chemical principles and many facets of force-field methodology.

## Figures and Tables

**Table 1:** Total natural charge *Q*_i_ and *q/t/d* coordination type for each water monomer (centered on O(*i*)) of the ^4,4,6^W_16_ cluster. (Similar tables are found in SI for each ^q,t,d^W_n_ cluster of the present work).

Cluster	O_i_	*Q*_i_	*q*/*t*/*d*
**^4,4,6^W_14_**	1	−0.00662	*t*_d_
	4	−0.00324	*d*
	7	+0.00963	*t*_a_
	10	−0.00636	*q*
	13	−0.00107	*q*
	16	−0.00414	*q*
	19	+0.00264	*q*
	22	+0.01427	*t*_a_
	25	+0.00079	*d*
	28	−0.00357	*d*
	31	+0.00465	*d*
	34	−0.00250	*d*
	37	+0.00087	*d*
	40	−0.00536	*t*_d_

**Table 2:** NRT bond orders *b*_ij_ and bond lengths *R*_ij_ (Å) for all O(*i*)···O(*j*) H-bonds of the ^4,4,6^W_14_ cluster (with atom numberings as shown in Figure 3). (See SI for similar tables for all clusters of the present work).

^4,4,6^W_14_	*i*	*j*	*b*_ij_	*R*_ij_
	1	4	0.1223	2.6972
	1	10	0.0638	2.8562
	1	22	0.0495	2.9173
	4	7	0.1301	2.7127
	7	10	0.0655	2.8861
	7	40	0.0490	2.9332
	10	13	0.1148	2.7789
	13	16	0.0923	2.7808
	13	40	0.1148	2.6940
	16	19	0.0937	2.8005
	16	25	0.0901	2.7772
	16	34	0.0923	2.7725
	19	28	0.0997	2.7415
	19	31	0.0984	2.7440
	22	37	0.0666	2.8167
	25	28	0.0839	2.7599
	31	34	0.0900	2.7594
	37	40	0.0616	2.8367

## Data Availability

Not applicable.

## Supplementary Materials

The following supporting information can be downloaded at: https://www.mdpi.com/article/10.3390/molecules27134218/s1. The Supporting Information (SI) file contains optimized geometrical coordinates, NBO/NRT keyword input, and other computational details in ready-to-run Gaussian input files for all equilibrium water clusters described in the paper. The file also contains tables of computed natural atomic charges and natural bond orders for all water clusters of the work.

## Sample Availability

Samples of the clusters are not available from the author.
