# Supplementary material for: High-Density “Windowpane” Coordination Patterns of Water Clusters and Their NBO/NRT Characterization
Source: Molecules. 2022 Jun 30;27(13):4218. doi: 10.3390/molecules27134218 (PMC9268199; doi:10.3390/molecules27134218)
Supplement: Supplementary file 1 [file molecules-27-04218-s001.zip › molecules-1733418-supplementary.pdf]

**SUPPLEMENTARY INFORMATION for "High-Density 'Windowpane' Coordination Patterns of Water Clusters and their NBO/NRT Characterization"**

F. Weinhold*

*Theoretical Chemistry Institute and Department of Chemistry, University of Wisconsin-Madison, Madison, Wisconsin 53706 (weinhold@chem.wisc.edu)*

This Supplementary Information (SI) consists of three sections:

(i) **NPA Charge Distributions** (pp. 2-7), displaying the natural atomic charge ($Q_A$) values for all monomer units of all windowpane clusters ${}^{q,t,d}W_n$, in tables similar to Table 1 of the main text;

(ii) **NRT Bond Order Distributions** (pp. 8-15), displaying the NRT bond orders $\{b_{O\cdots O}\}$ and associated $R_{O\cdots O}$ distances (Å) for all O–H···O hydrogen bonds of all windowpane clusters ${}^{q,t,d}W_n$, in tables similar to Table 2 of the main text;

(iii) **Optimized Cluster Geometries** (pp. 16-33), displaying ready-to-run *Gaussian-16* input files containing the optimized geometry, vibrational frequencies, and other thermochemical data for all windowpane clusters ${}^{q,t,d}W_n$, together with NBO keyword input to obtain the NRT bond orders and other NBO-based descriptors.

## NPA Charge Distributions

| cluster | $O_i$ | $Q_i$ | q/t/d |
|---|---|---|---|
| $^{0,2,4}W_6$ | 1 | –0.00815 | $t_d$ |
| | 4 | +0.00247 | d |
| | 7 | –0.00212 | d |
| | 10 | +0.00797 | $t_a$ |
| | 13 | –0.00238 | d |
| | 16 | +0.00222 | d |

| cluster | $O_i$ | $Q_i$ | q/t/d |
|---|---|---|---|
| $^{1,0,6}W_7$ | 1 | –0.00095 | d |
| | 4 | +0.00108 | d |
| | 7 | –0.00047 | d |
| | 10 | +0.00396 | q |
| | 13 | –0.00206 | d |
| | 16 | –0.00262 | d |
| | 19 | +0.00106 | d |

| cluster | $O_i$ | $Q_i$ | q/t/d |
|---|---|---|---|
| $^{0,4,4}W_8$ | 1 | –0.00710 | $t_d$ |
| | 4 | +0.00285 | d |
| | 7 | –0.00240 | d |
| | 10 | +0.00619 | $t_a$ |
| | 13 | +0.00247 | $t_a$ |
| | 16 | –0.00119 | $t_d$ |
| | 19 | –0.00347 | d |
| | 22 | +0.00262 | d |

| cluster | $O_i$ | $Q_i$ | q/t/d |
|---|---|---|---|
| $^{0,8,0}W_8$ | 1 | –0.01797 | $t_d$ |
| | 4 | +0.01787 | $t_a$ |
| | 7 | –0.01799 | $t_d$ |
| | 10 | +0.01801 | $t_a$ |
| | 13 | –0.01796 | $t_d$ |
| | 16 | +0.01799 | $t_a$ |
| | 19 | –0.01806 | $t_d$ |
| | 22 | +0.01811 | $t_a$ |

| cluster | $O_i$ | $Q_i$ | *q/t/d* |
|---|---|---|---|
| $^{1,2,5}W_8$ | 1 | –0.00580 | $t_d$ |
| | 4 | +0.00147 | *d* |
| | 7 | +0.00276 | *d* |
| | 10 | –0.00963 | *q* |
| | 13 | +0.01800 | $t_a$ |
| | 16 | –0.00314 | *d* |
| | 19 | –0.00293 | *d* |
| | 22 | –0.00073 | *d* |

| cluster | $O_i$ | $Q_i$ | *q/t/d* |
|---|---|---|---|
| $^{1,4,4}W_9$ | 1 | +0.00799 | $t_d$ |
| | 4 | +0.00246 | *d* |
| | 7 | –0.00818 | *d* |
| | 10 | –0.01029 | *q* |
| | 13 | +0.02714 | $t_d$ |
| | 16 | –0.00519 | *d* |
| | 19 | –0.00536 | $t_d$ |
| | 22 | –0.00070 | *d* |
| | 25 | –0.00786 | *d* |

| cluster | $O_i$ | $Q_i$ | *q/t/d* |
|---|---|---|---|
| $\mathbf{^{0,6,4}W_{10}}$ | 1 | –0.00682 | $t_d$ |
| | 4 | +0.00278 | *d* |
| | 7 | –0.00227 | *d* |
| | 10 | +0.00526 | $t_a$ |
| | 13 | +0.00056 | $t_a$ |
| | 16 | +0.00093 | $t_d$ |
| | 19 | –0.00002 | $t_a$ |
| | 22 | +0.00036 | $t_d$ |
| | 25 | –0.00358 | *d* |
| | 28 | +0.00278 | *d* |

| cluster | $O_i$ | $Q_i$ | *q/t/d* |
|---|---|---|---|
| $\mathbf{^{1,4,5}W_{10}}$ | 1 | –0.00493 | $t_d$ |
| | 4 | +0.00107 | *d* |
| | 7 | +0.00319 | *d* |
| | 10 | –0.01207 | *q* |
| | 13 | +0.02045 | $t_a$ |

| | | | |
|---|---|---|---|
| | 16 | +0.00393 | $t_a$ |
| | 19 | –0.00788 | $t_d$ |
| | 22 | –0.00152 | *d* |
| | 25 | –0.00093 | *d* |
| | 28 | –0.00132 | *d* |

| **cluster** | **O<sub></sub>i** | ***Q*i** | ***q/t/d*** |
|---|---|---|---|
| **$^{3,2,7}W_{12}$** | 1 | –0.00067 | $t_d$ |
| | 4 | +0.00131 | *d* |
| | 7 | +0.00160 | *d* |
| | 10 | –0.00667 | *q* |
| | 13 | +0.01490 | $t_a$ |
| | 16 | –0.00695 | *q* |
| | 19 | –0.00017 | *q* |
| | 22 | +0.00004 | *d* |
| | 25 | +0.00352 | *d* |
| | 28 | –0.00444 | *d* |
| | 31 | +0.00131 | *d* |
| | 34 | –0.00378 | *d* |

| **cluster** | **$O_i$** | **$Q_i$** | ***q/t/d*** |
|---|---|---|---|
| **$^{4,8,0}W_{12}$** | 1 | –0.01740 | $t_d$ |
| | 4 | +0.02204 | $t_a$ |
| | 7 | –0.01729 | $t_d$ |
| | 10 | +0.02202 | $t_a$ |
| | 13 | –0.00472 | *q* |
| | 16 | –0.00457 | *q* |
| | 19 | –0.00466 | *q* |
| | 22 | –0.00494 | *q* |
| | 25 | –0.01743 | $t_d$ |
| | 28 | +0.02210 | $t_a$ |
| | 31 | –0.01739 | $t_d$ |
| | 34 | +0.02222 | $t_a$ |

| **cluster** | **$O_i$** | **$Q_i$** | ***q/t/d*** |
|---|---|---|---|
| **$^{3,8,3}W_{14}$** | 1 | +0.01895 | $t_a$ |
| | 4 | +0.00079 | $t_d$ |
| | 7 | –0.01597 | $t_d$ |
| | 10 | –0.01309 | $t_d$ |

| | | | |
|---|---|---|---|
| | 13 | +0.01075 | *q* |
| | 16 | +0.00979 | *q* |
| | 19 | –0.00828 | *q* |
| | 22 | +0.00013 | $t_a$ |
| | 25 | +0.00566 | $t_a$ |
| | 28 | –0.00950 | *d* |
| | 31 | –0.00404 | *d* |
| | 34 | –0.00842 | *d* |
| | 37 | +0.01367 | $t_a$ |
| | 40 | –0.00046 | $t_d$ |

| **cluster** | **$O_i$** | **$Q_i$** | ***q/t/d*** |
|---|---|---|---|
| **$^{4,4,6}W_{14}$** | 1 | –0.00662 | $t_d$ |
| | 4 | –0.00324 | *d* |
| | 7 | +0.00963 | $t_a$ |
| | 10 | –0.00636 | *q* |
| | 13 | –0.00107 | *q* |
| | 16 | –0.00414 | *q* |
| | 19 | +0.00264 | *q* |
| | 22 | +0.01427 | $t_a$ |
| | 25 | +0.00079 | *d* |
| | 28 | –0.00357 | *d* |
| | 31 | +0.00465 | *d* |
| | 34 | –0.00250 | *d* |
| | 37 | +0.00087 | *d* |
| | 40 | –0.00536 | $t_d$ |

| **cluster** | **$O_i$** | **$Q_i$** | ***q/t/d*** |
|---|---|---|---|
| **$^{4,12,0}W_{16}$** | 1 | +0.01923 | $t_a$ |
| | 4 | +0.00054 | $t_d$ |
| | 7 | –0.01434 | $t_d$ |
| | 10 | –0.01171 | $t_a$ |
| | 13 | +0.00938 | *q* |
| | 16 | +0.00772 | *q* |
| | 19 | +0.00288 | *q* |
| | 22 | –0.01084 | *q* |
| | 25 | +0.00423 | $t_a$ |
| | 28 | +0.00230 | $t_a$ |
| | 31 | –0.03123 | $t_d$ |

| | | | |
|---|---|---|---|
| | 34 | +0.01684 | $t_a$ |
| | 37 | +0.01538 | $t_a$ |
| | 40 | +0.00045 | $t_d$ |
| | 43 | –0.02058 | $t_d$ |
| | 46 | +0.00975 | $t_a$ |

| **cluster** | **O$_i$** | ***Q*$_i$** | ***q/t/d*** |
|---|---|---|---|
| **$^{8,0,8}W_{16}$** | 1 | +0.00680 | *q* |
| | 4 | –0.00077 | *d* |
| | 7 | +0.00329 | *d* |
| | 10 | –0.00751 | *q* |
| | 13 | –0.00944 | *q* |
| | 16 | –0.01028 | *q* |
| | 19 | +0.00297 | *q* |
| | 22 | +0.01152 | *q* |
| | 25 | –0.00054 | *d* |
| | 28 | +0.00240 | *d* |
| | 31 | –0.00893 | *q* |
| | 34 | +0.00774 | *q* |
| | 37 | +0.00230 | *d* |
| | 40 | –0.00207 | *d* |
| | 43 | –0.00058 | *d* |
| | 46 | +0.00311 | *d* |

| **cluster** | **O$_i$** | ***Q*$_i$** | ***q/t/d*** |
|---|---|---|---|
| **$^{8,8,0}W_{16}$** | 1 | –0.01708 | $t_d$ |
| | 4 | +0.02232 | $t_a$ |
| | 7 | –0.01718 | $t_d$ |
| | 10 | +0.02234 | $t_a$ |
| | 13 | +0.00000 | *q* |
| | 16 | –0.00517 | *q* |
| | 19 | –0.00005 | *q* |
| | 22 | –0.00524 | *q* |
| | 25 | –0.00530 | *q* |
| | 28 | –0.00010 | *q* |
| | 31 | –0.00530 | *q* |
| | 34 | –0.00007 | *q* |
| | 37 | –0.01706 | $t_d$ |
| | 40 | +0.02240 | $t_a$ |

| | | | |
|---|---|---|---|
| | 43 | –0.01700 | $t_d$ |
| | 46 | +0.02240 | $t_a$ |

# NRT Bond Order Distributions

| $^{0,0,4}W_4$ | *i* | *j* | $b_{ij}$ | $R_{ij}$ |
|---|---|---|---|---|
| | 1 | 4 | 0.1068 | 2.7401 |
| | 1 | 10 | 0.1068 | 2.7401 |
| | 4 | 7 | 0.1068 | 2.7401 |
| | 7 | 10 | 0.1068 | 2.7401 |

| $^{0,2,4}W_6$ | *i* | *j* | $b_{ij}$ | $R_{ij}$ |
|---|---|---|---|---|
| | 1 | 4 | 0.1365 | 2.6920 |
| | 1 | 10 | 0.0389 | 2.9572 |
| | 1 | 16 | 0.0813 | 2.7886 |
| | 4 | 7 | 0.1302 | 2.6957 |
| | 7 | 10 | 0.1351 | 2.6953 |
| | 10 | 13 | 0.0804 | 2.8021 |
| | 13 | 16 | 0.0873 | 2.7721 |

| $^{1,0,6}W_7$ | *i* | *j* | $b_{ij}$ | $R_{ij}$ |
|---|---|---|---|---|
| | 1 | 4 | 0.1061 | 2.7412 |
| | 1 | 10 | 0.0766 | 2.7513 |
| | 4 | 7 | 0.1041 | 2.7377 |
| | 7 | 10 | 0.0706 | 2.7570 |
| | 10 | 13 | 0.0637 | 2.7513 |
| | 10 | 19 | 0.0695 | 2.7677 |
| | 13 | 16 | 0.0999 | 2.7469 |
| | 16 | 19 | 0.1082 | 2.7349 |

| $^{0,4,4}W_8$ | *i* | *j* | $b_{ij}$ | $R_{ij}$ |
|---|---|---|---|---|
| | 1 | 4 | 0.1462 | 2.6785 |
| | 1 | 10 | 0.0280 | 3.0224 |
| | 1 | 16 | 0.1052 | 2.7426 |
| | 4 | 7 | 0.1387 | 2.6792 |
| | 7 | 10 | 0.1440 | 2.6812 |
| | 10 | 13 | 0.1063 | 2.7521 |
| | 13 | 16 | 0.0322 | 2.9885 |
| | 13 | 19 | 0.0724 | 2.8228 |
| | 16 | 22 | 0.0750 | 2.8075 |
| | 19 | 22 | 0.0822 | 2.7822 |

| $^{0,8,0}W_8$ | *i* | *j* | $b_{ij}$ | $R_{ij}$ |
|---|---|---|---|---|
| | 1 | 4 | 0.1533 | 2.6849 |
| | 1 | 10 | 0.0271 | 2.8605 |
| | 1 | 16 | 0.0261 | 2.8601 |
| | 4 | 7 | 0.0283 | 2.8596 |
| | 4 | 19 | 0.0247 | 2.8603 |
| | 7 | 10 | 0.1534 | 2.6846 |
| | 7 | 22 | 0.0283 | 2.8600 |
| | 10 | 13 | 0.0267 | 2.8597 |
| | 13 | 16 | 0.0276 | 2.8599 |
| | 13 | 22 | 0.1535 | 2.6845 |
| | 16 | 19 | 0.1534 | 2.6845 |
| | 19 | 22 | 0.0247 | 2.8606 |

| $^{1,2,5}W_8$ | *i* | *j* | $b_{ij}$ | $R_{ij}$ |
|---|---|---|---|---|
| | 1 | 4 | 0.1259 | 2.6991 |
| | 1 | 10 | 0.0499 | 2.8988 |
| | 1 | 22 | 0.0633 | 2.8435 |
| | 4 | 7 | 0.1225 | 2.7101 |
| | 7 | 10 | 0.1136 | 2.7477 |
| | 10 | 13 | 0.1765 | 2.6666 |
| | 10 | 19 | 0.0870 | 2.7837 |
| | 13 | 16 | 0.0761 | 2.7990 |
| | 13 | 22 | 0.0609 | 2.8239 |
| | 16 | 19 | 0.0865 | 2.7775 |

| $^{1,4,4}W_9$ | *i* | *j* | $b_{ij}$ | $R_{ij}$ |
|---|---|---|---|---|
| | 1 | 4 | 0.0942 | 2.7519 |
| | 1 | 10 | 0.0775 | 2.8377 |
| | 1 | 22 | 0.0318 | 2.8918 |
| | 4 | 7 | 0.0797 | 2.8139 |
| | 7 | 10 | 0.1563 | 2.6300 |
| | 7 | 25 | 0.0401 | 2.9047 |
| | 10 | 13 | 0.1783 | 2.6516 |
| | 10 | 19 | 0.0722 | 2.8307 |
| | 13 | 16 | 0.0757 | 2.8002 |
| | 13 | 22 | 0.0377 | 2.8904 |
| | 16 | 19 | 0.0903 | 2.7557 |
| | 19 | 25 | 0.0228 | 2.9178 |

| $^{0,6,4}$**W**$_{10}$ | ***i*** | ***j*** | $b_{ij}$ | $R_{ij}$ |
|---|---|---|---|---|
| | 1 | 4 | 0.1489 | 2.6746 |
| | 1 | 10 | 0.0257 | 3.0373 |
| | 1 | 16 | 0.1112 | 2.7333 |
| | 4 | 7 | 0.1417 | 2.6740 |
| | 7 | 10 | 0.1465 | 2.6755 |
| | 10 | 13 | 0.1137 | 2.7380 |
| | 13 | 16 | 0.0230 | 3.0541 |
| | 13 | 19 | 0.0948 | 2.7733 |
| | 16 | 22 | 0.0963 | 2.7642 |
| | 19 | 22 | 0.0303 | 2.9971 |
| | 19 | 25 | 0.0695 | 2.8306 |
| | 22 | 28 | 0.0720 | 2.8123 |
| | 25 | 28 | 0.0797 | 2.7870 |

| $^{1,4,5}$**W**$_{10}$ | ***i*** | ***j*** | $b_{ij}$ | $R_{ij}$ |
|---|---|---|---|---|
| | 1 | 4 | 0.1238 | 2.6994 |
| | 1 | 10 | 0.0481 | 2.8857 |
| | 1 | 22 | 0.0552 | 2.8548 |
| | 4 | 7 | 0.1210 | 2.7156 |
| | 7 | 10 | 0.0998 | 2.7564 |
| | 10 | 13 | 0.2042 | 2.6307 |
| | 10 | 19 | 0.1275 | 2.7273 |
| | 13 | 16 | 0.1079 | 2.7462 |
| | 16 | 19 | 0.0308 | 2.9976 |
| | 16 | 25 | 0.0723 | 2.8257 |
| | 19 | 28 | 0.0792 | 2.7950 |
| | 25 | 28 | 0.0748 | 2.7913 |

| $^{3,2,7}$**W**$_{12}$ | ***i*** | ***j*** | $b_{ij}$ | $R_{ij}$ |
|---|---|---|---|---|
| | 1 | 4 | 0.1219 | 2.7018 |
| | 1 | 10 | 0.0676 | 2.8712 |
| | 1 | 22 | 0.0526 | 2.8394 |
| | 4 | 7 | 0.1188 | 2.7178 |
| | 7 | 10 | 0.1155 | 2.7455 |
| | 10 | 13 | 0.1596 | 2.691 |
| | 10 | 19 | 0.0904 | 2.7886 |

| | | | | |
|---|---|---|---|---|
| | 13 | 22 | 0.0649 | 2.8186 |
| | 13 | 16 | 0.0685 | 2.8446 |
| | 16 | 25 | 0.0720 | 2.7863 |
| | 16 | 34 | 0.1012 | 2.7519 |
| | 19 | 28 | 0.0728 | 2.7439 |
| | 19 | 31 | 0.0944 | 2.7560 |
| | 25 | 28 | 0.0791 | 2.7684 |
| | 31 | 34 | 0.0958 | 2.7531 |

–

| $^{3,4,5}$**W$_{12}$** | ***i*** | ***j*** | ***b*$_{ij}$** | ***R*$_{ij}$** |
|---|---|---|---|---|
| | 1 | 4 | 0.0718 | 2.7944 |
| | 1 | 10 | 0.0877 | 2.7604 |
| | 4 | 7 | 0.1611 | 2.6749 |
| | 4 | 13 | 0.0718 | 2.8632 |
| | 7 | 16 | 0.0264 | 2.8826 |
| | 7 | 22 | 0.0561 | 2.8650 |
| | 10 | 13 | 0.0967 | 2.7489 |
| | 13 | 16 | 0.1108 | 2.7557 |
| | 13 | 19 | 0.0579 | 2.8272 |
| | 16 | 22 | 0.0436 | 2.8720 |
| | 19 | 22 | 0.1052 | 2.7520 |
| | 19 | 25 | 0.0498 | 2.8903 |
| | 19 | 31 | 0.1104 | 2.7418 |
| | 22 | 28 | 0.0772 | 2.8009 |
| | 25 | 28 | 0.0647 | 2.8362 |
| | 25 | 34 | 0.1348 | 2.7007 |
| | 31 | 34 | 0.1188 | 2.7178 |

| $^{4,8,0}$**W$_{12}$** | ***i*** | ***j*** | ***b*$_{ij}$** | ***R*$_{ij}$** |
|---|---|---|---|---|
| | 1 | 4 | 0.1475 | 2.6949 |
| | 1 | 10 | 0.0561 | 2.8813 |
| | 1 | 16 | 0.0538 | 2.8392 |
| | 4 | 7 | 0.0560 | 2.8820 |
| | 4 | 19 | 0.0394 | 2.8570 |
| | 7 | 10 | 0.1475 | 2.6947 |
| | 7 | 22 | 0.0541 | 2.8380 |
| | 10 | 13 | 0.0394 | 2.8575 |
| | 13 | 16 | 0.1082 | 2.7784 |

| | | | | |
|---|---|---|---|---|
| | 13 | 22 | 0.1078 | 2.7795 |
| | 13 | 25 | 0.0542 | 2.8380 |
| | 16 | 19 | 0.1081 | 2.7785 |
| | 16 | 28 | 0.0395 | 2.8572 |
| | 19 | 22 | 0.1079 | 2.7789 |
| | 19 | 31 | 0.0538 | 2.8384 |
| | 22 | 34 | 0.0392 | 2.8585 |
| | 25 | 28 | 0.1478 | 2.6943 |
| | 25 | 34 | 0.0561 | 2.8831 |
| | 28 | 31 | 0.0560 | 2.8826 |

| $^{3,8,3}W_{14}$ | ***i*** | ***j*** | $b_{ij}$ | $R_{ij}$ |
|---|---|---|---|---|
| | 1 | 4 | 0.0479 | 2.8915 |
| | 1 | 10 | 0.1394 | 2.6919 |
| | 1 | 40 | 0.0513 | 2.8748 |
| | 4 | 7 | 0.0832 | 2.7877 |
| | 4 | 13 | 0.0480 | 2.8933 |
| | 7 | 16 | 0.1956 | 2.6317 |
| | 7 | 40 | 0.0832 | 2.7941 |
| | 10 | 13 | 0.0652 | 2.8334 |
| | 10 | 37 | 0.0475 | 2.9123 |
| | 13 | 19 | 0.0456 | 2.8578 |
| | 16 | 22 | 0.0345 | 2.9366 |
| | 19 | 22 | 0.0674 | 2.8305 |
| | 19 | 25 | 0.0640 | 2.8759 |
| | 19 | 31 | 0.1063 | 2.7518 |
| | 22 | 28 | 0.1017 | 2.7621 |
| | 25 | 28 | 0.0752 | 2.8198 |
| | 25 | 34 | 0.1410 | 2.6906 |

| $^{4,4,6}W_{14}$ | ***i*** | ***j*** | $b_{ij}$ | $R_{ij}$ |
|---|---|---|---|---|
| | 1 | 4 | 0.1223 | 2.6972 |
| | 1 | 10 | 0.0638 | 2.8562 |
| | 1 | 22 | 0.0495 | 2.9173 |
| | 4 | 7 | 0.1301 | 2.7127 |
| | 7 | 10 | 0.0655 | 2.8861 |
| | 7 | 40 | 0.0490 | 2.9332 |
| | 10 | 13 | 0.1148 | 2.7789 |
| | 13 | 16 | 0.0923 | 2.7808 |

| | | | | |
|---|---|---|---|---|
| | 13 | 40 | 0.1148 | 2.6940 |
| | 16 | 19 | 0.0937 | 2.8005 |
| | 16 | 25 | 0.0901 | 2.7772 |
| | 16 | 34 | 0.0923 | 2.7725 |
| | 19 | 28 | 0.0997 | 2.7415 |
| | 19 | 31 | 0.0984 | 2.7440 |
| | 22 | 37 | 0.0666 | 2.8167 |
| | 25 | 28 | 0.0839 | 2.7599 |
| | 31 | 34 | 0.0900 | 2.7594 |
| | 37 | 40 | 0.0616 | 2.8367 |

| $^{4,12,0}W_{16}$ | $i$ | $j$ | $b_{ij}$ | $R_{ij}$ |
|---|---|---|---|---|
| | 1 | 4 | 0.0460 | 2.8980 |
| | 1 | 10 | 0.1399 | 2.6902 |
| | 1 | 40 | 0.0501 | 2.8739 |
| | 4 | 7 | 0.0819 | 2.7894 |
| | 4 | 13 | 0.0492 | 2.8935 |
| | 7 | 16 | 0.1893 | 2.6394 |
| | 7 | 40 | 0.0819 | 2.7994 |
| | 10 | 13 | 0.0710 | 2.8210 |
| | 10 | 37 | 0.0461 | 2.9147 |
| | 13 | 19 | 0.0481 | 2.8574 |
| | 16 | 22 | 0.0379 | 2.9047 |
| | 16 | 37 | 0.1127 | 2.7430 |
| | 19 | 22 | 0.1045 | 2.7846 |
| | 19 | 25 | 0.0288 | 2.9592 |
| | 19 | 31 | 0.0768 | 2.7963 |
| | 22 | 28 | 0.0447 | 2.8969 |
| | 22 | 46 | 0.0739 | 2.8280 |
| | 25 | 28 | 0.0855 | 2.8269 |
| | 25 | 34 | 0.1175 | 2.7624 |
| | 28 | 43 | 0.0437 | 2.9257 |
| | 31 | 34 | 0.1998 | 2.6290 |
| | 31 | 46 | 0.0509 | 2.8990 |
| | 34 | 43 | 0.0484 | 2.8842 |
| | 37 | 40 | 0.0354 | 2.9494 |
| | 43 | 46 | 0.1407 | 2.7047 |

| $^{8,0,8}W_{16}$ | *i* | *j* | $b_{ij}$ | $R_{ij}$ |
|---|---|---|---|---|
| | 1 | 4 | 0.0951 | 2.7445 |
| | 1 | 10 | 0.1161 | 2.7665 |
| | 1 | 22 | 0.0691 | 2.7757 |
| | 1 | 31 | 0.0819 | 2.7822 |
| | 4 | 7 | 0.0975 | 2.7630 |
| | 7 | 10 | 0.0690 | 2.7731 |
| | 10 | 13 | 0.0707 | 2.8545 |
| | 10 | 19 | 0.0846 | 2.8208 |
| | 13 | 16 | 0.0818 | 2.8054 |
| | 13 | 22 | 0.1076 | 2.7763 |
| | 13 | 40 | 0.0901 | 2.7615 |
| | 16 | 19 | 0.1005 | 2.8240 |
| | 16 | 25 | 0.0842 | 2.7734 |
| | 16 | 34 | 0.0789 | 2.7921 |
| | 19 | 28 | 0.0759 | 2.7644 |
| | 19 | 31 | 0.0890 | 2.7870 |
| | 22 | 34 | 0.0778 | 2.7782 |
| | 22 | 37 | 0.0942 | 2.7332 |
| | 25 | 28 | 0.0896 | 2.7629 |
| | 31 | 34 | 0.1009 | 2.8178 |
| | 31 | 43 | 0.0854 | 2.7708 |
| | 34 | 46 | 0.0780 | 2.7668 |
| | 37 | 40 | 0.0862 | 2.7714 |
| | 43 | 46 | 0.0911 | 2.7610 |

| $^{8,8,0}W_{16}$ | *i* | *j* | $b_{ij}$ | $R_{ij}$ |
|---|---|---|---|---|
| | 1 | 4 | 0.1494 | 2.6919 |
| | 1 | 10 | 0.0347 | 2.8823 |
| | 1 | 16 | 0.0409 | 2.8353 |
| | 4 | 7 | 0.0429 | 2.8823 |
| | 4 | 19 | 0.0405 | 2.8562 |
| | 7 | 10 | 0.1494 | 2.6919 |
| | 7 | 22 | 0.0411 | 2.8354 |
| | 10 | 13 | 0.0405 | 2.8560 |
| | 13 | 16 | 0.0997 | 2.7928 |
| | 13 | 22 | 0.1050 | 2.7922 |
| | 13 | 25 | 0.0387 | 2.8358 |
| | 16 | 19 | 0.1050 | 2.7921 |

| | | | | |
|---|---|---|---|---|
| | 16 | 28 | 0.0390 | 2.8358 |
| | 19 | 22 | 0.0997 | 2.7929 |
| | 19 | 31 | 0.0389 | 2.8354 |
| | 22 | 34 | 0.0387 | 2.8362 |
| | 25 | 28 | 0.1005 | 2.7929 |
| | 25 | 34 | 0.1053 | 2.7912 |
| | 25 | 37 | 0.0371 | 2.8353 |
| | 28 | 31 | 0.1049 | 2.7922 |
| | 28 | 46 | 0.0405 | 2.8578 |
| | 31 | 43 | 0.0367 | 2.8352 |
| | 34 | 40 | 0.0405 | 2.8561 |
| | 37 | 40 | 0.1495 | 2.6920 |
| | 37 | 46 | 0.0342 | 2.8825 |
| | 40 | 43 | 0.0336 | 2.8830 |
| | 43 | 46 | 0.1492 | 2.6925 |

# Optimized Cluster Geometries

```
%nproc=8
%chk=w2
%mem=2GB
#B3LYP/6-311++G** POP=NBO6Read

water dimer, E(RB3LYP) =  -152.926350055 (dE = -5.83 kcal/mol)
nu: 144,165,175,...
Vibrational temperatures:    206.49   237.01   251.99   283.88   534.25
         (Kelvin)            968.26  2320.93  2344.01  5331.39  5488.10
                            5600.08  5632.57
Zero-point correction=                           0.046234 (Hartree/Particle)
Thermal correction to Energy=                    0.051973
Thermal correction to Enthalpy=                  0.052917
Thermal correction to Gibbs Free Energy=         0.020146
Sum of electronic and zero-point Energies=           -152.880116
Sum of electronic and thermal Energies=              -152.874377
Sum of electronic and thermal Enthalpies=            -152.873433
Sum of electronic and thermal Free Energies=         -152.906204

  0  1
 H
 O     1   0.9681
 H     2   0.9775    1    105.8230
 O     3   1.9059    2    171.3648    1    180.0262
 H     4   0.9698    3    109.7952    2     57.8695
 H     4   0.9698    3    109.7952    2    302.0524

$NBO file=water_dimer archive dipole nrt plot $END
```

```
**************************************************************************************
%mem=2gb
%nprocshared=8
%chk=w4c
#b3lyp/6-311++G** pop=nbo7read

w4c, E(RB3LYP) =  -305.883182879
nu: 56,92,212,...
 Vibrational temperatures:     80.50   132.86   305.24   315.75   353.06
          (Kelvin)            353.07   373.97   377.79   377.80   447.25
                              594.34   642.43   674.69   674.71  1138.90
                             1214.13  1214.13  1461.70  2346.80  2371.02
                             2371.03  2418.89  4856.35  4991.12  4991.13
                             5045.49  5588.27  5589.36  5589.36  5590.89
 Zero-point correction=                           0.098934 (Hartree/Particle)
 Thermal correction to Energy=                    0.108534
 Thermal correction to Enthalpy=                  0.109478
 Thermal correction to Gibbs Free Energy=         0.065929
 Sum of electronic and zero-point Energies=           -305.784248
 Sum of electronic and thermal Energies=              -305.774649
 Sum of electronic and thermal Enthalpies=            -305.773704
 Sum of electronic and thermal Free Energies=         -305.817254

0 1
               8         -0.043224    1.936641    0.028239
               1         -0.824062    1.339305   -0.004245
               1         -0.196073    2.541087    0.760117
               8         -1.936652   -0.043224   -0.028238
               1         -1.339311   -0.824059    0.004247
               1         -2.541094   -0.196075   -0.760120
               8          0.043225   -1.936640    0.028239
               1          0.824061   -1.339300   -0.004243
               1          0.196076   -2.541087    0.760116
               8          1.936656    0.043224   -0.028235
```

```
               1          1.339308    0.824055    0.004252
               1          2.541050    0.196070   -0.760157

$nbo file=w4c archive nrt plot $end

**********************************************************************************
%mem=2gb
%nprocshared=8
%chk=w6_q0_t2_d4
#b3lyp/6-311++G** pop=nbo7read

w6_q0_t2_d4 ("2-pane"), E(RB3LYP) =  -458.831339373
nu: 32,44,62,...
 Vibrational temperatures:     46.06    62.70    89.36   112.32   141.90
          (Kelvin)            212.21   262.12   284.37   300.12   336.45
                              356.55   366.68   380.12   416.18   422.01
                              435.32   462.58   574.56   599.63   663.11
                              679.58   714.43   804.39   902.73  1085.48
                             1128.82  1238.68  1266.92  1330.31  1507.48
                             2343.30  2360.76  2364.51  2393.65  2420.78
                             2471.52  4696.19  4809.84  4887.12  5042.15
                             5126.96  5152.88  5356.38  5576.31  5588.87
                             5591.25  5591.77  5593.75
 Zero-point correction=                           0.149713 (Hartree/Particle)
 Thermal correction to Energy=                    0.164610
 Thermal correction to Enthalpy=                  0.165554
 Thermal correction to Gibbs Free Energy=         0.108550
 Sum of electronic and zero-point Energies=           -458.681627
 Sum of electronic and thermal Energies=              -458.666730
 Sum of electronic and thermal Enthalpies=            -458.665786
 Sum of electronic and thermal Free Energies=         -458.722789

   0  1
               8         -0.102847    1.546527    0.865219
               1         -0.954782    1.531691    0.359698
               1         -0.244471    2.119056    1.625438
               8         -2.429522    1.232050   -0.451817
               1         -2.526997    0.248670   -0.487527
               1         -2.610745    1.555085   -1.338990
               8         -2.336467   -1.462044   -0.437681
               1         -1.462734   -1.586579    0.010730
               1         -2.954645   -2.053823    0.000041
               8          0.015314   -1.408329    0.878002
               1          0.057314   -0.465780    1.104985
               1          0.864936   -1.587018    0.427081
               8          2.498662   -1.404974   -0.419934
               1          2.585015   -0.432334   -0.497755
               1          3.313569   -1.714925   -0.014822
               8          2.360444    1.363630   -0.429104
               1          1.494977    1.540541   -0.007352
               1          2.393883    1.910541   -1.219010

$nbo file=w6_q0_t2_d4 archive nrt plot $end

**********************************************************************************
%mem=2gb
%nprocshared=8
%chk=w8_q0_t4_d4
#b3lyp/6-311++G** pop=nbo7read

w8_q0_t4_d4 ("4-pane ladder"), E(RB3LYP) =  -611.777396076
nu: 20,29,44,...
 Vibrational temperatures:     28.53    41.47    63.82    70.85    73.77
          (Kelvin)            104.98   131.19   142.29   177.42   201.13
                              245.72   261.18   292.88   316.98   342.38
                              351.78   355.64   371.20   387.50   412.54
```

```
                              420.18   435.69   453.38   467.01   550.74
                              583.17   655.72   670.18   690.44   722.68
                              744.34   783.06   867.10   915.43  1040.48
                             1092.99  1210.04  1223.58  1241.15  1319.50
                             1370.98  1528.39  2341.44  2355.17  2361.50
                             2379.03  2401.28  2428.17  2448.10  2481.53
                             4627.88  4745.79  4835.93  4943.26  4990.94
                             5082.10  5163.22  5190.01  5399.34  5415.36
                             5574.19  5576.54  5588.85  5592.42  5592.47
                             5593.86
 Zero-point correction=                           0.200250 (Hartree/Particle)
 Thermal correction to Energy=                    0.220627
 Thermal correction to Enthalpy=                  0.221571
 Thermal correction to Gibbs Free Energy=         0.150633
 Sum of electronic and zero-point Energies=           -611.577146
 Sum of electronic and thermal Energies=              -611.556769
 Sum of electronic and thermal Enthalpies=            -611.555825
 Sum of electronic and thermal Free Energies=         -611.626764

   0  1
            8         1.215665    1.581900   -0.767618
            1         2.113017    1.506917   -0.348896
            1         1.317630    2.167450   -1.524278
            8         3.646856    1.172786    0.279516
            1         3.749541    0.187304    0.275238
            1         3.934382    1.472100    1.146682
            8         3.560690   -1.504197    0.212302
            1         2.642354   -1.619614   -0.144779
            1         4.127063   -2.096062   -0.290429
            8         1.095055   -1.437270   -0.838802
            1         1.017738   -0.503416   -1.082249
            1         0.303570   -1.606179   -0.279255
            8        -1.119606   -1.453350    0.794869
            1        -1.144067   -0.513638    1.028794
            1        -2.012160   -1.639640    0.444599
            8        -1.035024    1.533962    0.798895
            1        -0.221045    1.618999    0.251392
            1        -0.903556    2.090449    1.572856
            8        -3.758374   -1.467490   -0.207505
            1        -3.858477   -0.497125   -0.282184
            1        -4.515743   -1.777775    0.296793
            8        -3.618952    1.310353   -0.275865
            1        -2.713709    1.487740    0.047531
            1        -3.727018    1.828946   -1.078155

$nbo file=w8_q0_t4_d4 archive nrt plot $end

************************************************************************
%mem=2gb
%nprocshared=8
%chk=w10_q0_t6_d4
#b3lyp/6-311++G** pop=nbo7read

w10_q0_t6_d4 ("4-pane ladder"), E(RB3LYP) =  -764.722600374
nu: 12,20,30,...
 Vibrational temperatures:     17.36    28.17    42.85    60.96    61.79
          (Kelvin)             68.50    69.05   104.55   122.05   129.59
                              141.86   157.77   177.62   198.23   240.99
                              253.12   287.70   290.47   312.33   330.94
                              353.83   361.44   365.00   385.19   395.34
                              399.78   405.11   418.46   436.32   459.70
                              473.72   539.96   574.63   634.91   650.85
                              668.78   694.33   712.31   726.98   748.86
                              761.91   860.06   878.46   886.12  1020.98
                             1080.59  1147.25  1209.55  1221.09  1249.20
                             1268.50  1338.10  1389.34  1535.08  2339.94
```

```
                             2352.90  2359.32  2370.62  2390.23  2410.26
                             2426.33  2438.64  2463.28  2483.89  4605.94
                             4725.20  4818.23  4895.19  4940.75  5005.39
                             5054.06  5099.77  5178.77  5204.54  5416.77
                             5427.47  5450.79  5573.47  5575.30  5577.34
                             5588.82  5592.31  5593.03  5594.45
 Zero-point correction=                           0.250654 (Hartree/Particle)
 Thermal correction to Energy=                    0.276622
 Thermal correction to Enthalpy=                  0.277566
 Thermal correction to Gibbs Free Energy=         0.192293
 Sum of electronic and zero-point Energies=           -764.471946
 Sum of electronic and thermal Energies=              -764.445978
 Sum of electronic and thermal Enthalpies=            -764.445034
 Sum of electronic and thermal Free Energies=         -764.530307

   0  1
             8         -2.471232    1.568119    0.747501
             1         -3.318946    1.482375    0.236016
             1         -2.661100    2.151721    1.488549
             8         -4.777481    1.150454   -0.541081
             1         -4.886152    0.164924   -0.545500
             1         -4.980676    1.450039   -1.431684
             8         -4.715089   -1.521855   -0.467714
             1         -3.837792   -1.641738   -0.018955
             1         -5.331434   -2.115431   -0.030031
             8         -2.372818   -1.466510    0.826545
             1         -2.317059   -0.537597    1.091368
             1         -1.528093   -1.629696    0.345702
             8         -0.019568   -1.487801   -0.572809
             1          0.040171   -0.562465   -0.846399
             1          0.833040   -1.659679   -0.117701
             8         -0.081844    1.565700   -0.579869
             1         -0.946886    1.612113   -0.108637
             1         -0.172661    2.113243   -1.365969
             8          2.405067   -1.496081    0.773392
             1          2.455412   -0.557616    1.005576
             1          3.250483   -1.679235    0.320995
             8          2.312164    1.499418    0.800456
             1          1.451278    1.576590    0.332830
             1          2.242621    2.038427    1.594563
             8          4.931225   -1.494584   -0.503730
             1          5.034977   -0.523679   -0.556177
             1          5.728384   -1.828032   -0.082304
             8          4.808463    1.289569   -0.477628
             1          3.931187    1.461529   -0.083731
             1          4.862147    1.832771   -1.269015

$nbo file=w10_q0_t6_d4 archive nrt plot $end

********************************************************************************
%mem=2gb
%nprocshared=8
%chk=w9_q1_t4_d4
#b3lyp/6-311++G** pop=nbo7read opt freq

w9_4_pane (w9_q1_t4_d4 "saddle"), E(RB3LYP) =  -688.246307864
nu: 12,25,37,...
 Vibrational temperatures:     17.43    36.33    53.52    64.71    76.51
          (Kelvin)             89.50    99.32   123.19   136.20   143.81
                              175.14   212.81   235.56   254.25   273.68
                              283.67   287.03   316.11   323.58   327.82
                              341.22   375.10   387.72   405.36   438.77
                              461.79   495.23   539.46   567.19   612.22
                              629.80   665.78   692.62   767.62   772.45
                              791.03   811.41   956.57  1008.13  1035.62
                             1073.07  1118.50  1153.30  1178.36  1221.82
```

```
                           1250.36  1407.33  1568.05  2315.21  2352.42
                           2360.46  2370.21  2381.74  2387.02  2402.71
                           2412.35  2492.27  4443.79  4777.17  4877.14
                           4954.01  5006.41  5125.07  5175.52  5207.92
                           5259.38  5316.47  5373.55  5495.09  5562.42
                           5568.98  5573.84  5591.00  5596.27  5597.96
 Zero-point correction=                           0.225212 (Hartree/Parti$
 Thermal correction to Energy=                    0.248483
 Thermal correction to Enthalpy=                  0.249427
 Thermal correction to Gibbs Free Energy=         0.171104
 Sum of electronic and zero-point Energies=           -688.021096
 Sum of electronic and thermal Energies=              -687.997825
 Sum of electronic and thermal Enthalpies=            -687.996881
 Sum of electronic and thermal Free Energies=         -688.075204

   0  1
             8          0.131053   -2.498922   -1.052882
             1          1.078473   -2.594556   -0.807112
             1          0.110244   -2.388383   -2.009173
             8          2.738637   -2.306524   -0.194697
             1          2.824623   -1.449619    0.272634
             1          3.566700   -2.452947   -0.660400
             8          2.511496    0.146091    1.165794
             1          1.521992    0.091597    1.057778
             1          2.684216    0.101866    2.111960
             8         -0.009101   -0.100293    0.456886
             1         -0.055396   -0.898067   -0.101622
             1         -0.908061   -0.019878    0.882922
             8         -2.542058    0.055159    1.225616
             1         -2.850282    0.817597    0.695451
             1         -2.852951   -0.756293    0.791284
             8         -2.853785    2.350301   -0.348011
             1         -1.934415    2.522623   -0.647545
             1         -3.214294    3.197920   -0.074785
             8         -0.194190    2.278638   -1.066041
             1         -0.074543    1.414737   -0.629839
             1          0.649058    2.730016   -0.904888
             8         -2.570862   -2.551145   -0.023578
             1         -1.658023   -2.729908   -0.305110
             1         -2.906322   -3.376444    0.337663
             8          2.569675    2.693908   -0.228034
             1          2.703105    1.875183    0.282204
             1          3.068956    3.376846    0.228153

$nbo file=w9_q1_t4_d4 archive nrt plot thresh=1.4 $end

***************************************************************************
%mem=2gb
%nprocshared=8
%chk=w8_t8
#b3lyp/6-311++G** pop=nbo7read

w8_t8 ("cube"), E(RB3LYP) =  -611.792369303
nu: 81,83,85,...
 Vibrational temperatures:    116.05   119.09   122.16   124.99   125.05
          (Kelvin)            182.83   230.44   241.33   250.51   250.63
                              272.63   296.27   296.41   298.01   384.08
                              393.58   393.67   418.81   421.40   437.91
                              501.50   501.59   644.41   663.34   663.46
                              752.98   757.78   792.28   801.16   801.29
                              887.53  1008.12  1008.53  1014.50  1065.17
                             1065.27  1109.30  1284.20  1346.12  1516.02
                             1516.49  1616.43  2355.35  2356.90  2356.91
                             2374.79  2411.59  2411.71  2449.30  2480.70
                             4622.93  4657.52  4658.30  4744.90  5199.81
                             5199.92  5222.88  5227.18  5248.45  5248.70
```

```
                         5312.94  5312.97  5579.80  5579.96  5580.04
                         5580.50
 Zero-point correction=                           0.204049 (Hartree/Particle)
 Thermal correction to Energy=                    0.222157
 Thermal correction to Enthalpy=                  0.223101
 Thermal correction to Gibbs Free Energy=         0.161017
 Sum of electronic and zero-point Energies=           -611.588320
 Sum of electronic and thermal Energies=              -611.570213
 Sum of electronic and thermal Enthalpies=            -611.569268
 Sum of electronic and thermal Free Energies=         -611.631352

   0  1
             8          2.082972    0.137875   -1.345066
             1          2.178068    0.140096   -0.355425
             1          2.968129    0.194557   -1.717317
             8          1.952156    0.119848    1.336558
             1          1.346677    0.853935    1.546015
             1          1.446927   -0.687727    1.541240
             8         -0.137979    2.071318    1.359495
             1         -0.140516    2.174413    0.370574
             1         -0.193407    2.953527    1.738862
             8         -0.119835    1.962628   -1.322889
             1          0.687908    1.459142   -1.531057
             1         -0.853817    1.358315   -1.536110
             8         -2.071050   -0.127565   -1.361248
             1         -2.174732   -0.138096   -0.372445
             1         -2.952894   -0.181221   -1.741711
             8          0.130806   -1.952417   -1.334686
             1         -0.674714   -1.446603   -1.545973
             1          0.867076   -1.347821   -1.538817
             8          0.126437   -2.081968    1.346666
             1          0.137754   -2.177313    0.357046
             1          0.182379   -2.966759    1.719867
             8         -1.964035   -0.129493    1.321120
             1         -1.459143    0.675789    1.535493
             1         -1.361471   -0.866040    1.530151

$nbo file=w8_t8 archive nrt plot $end

************************************************************************
%mem=2gb
%nprocshared=8
%chk=w12_q4_t8
#b3lyp/6-311++G** pop=nbo7read

w12_q4_t8 ("2-cube"), E(RB3LYP) = -917.699234818
 nu: 59,61(2),78(2),...
 Vibrational temperatures:     84.29    87.48    87.49   112.07   112.09
          (Kelvin)            124.64   129.42   133.17   156.70   170.63
                              176.95   189.18   189.37   227.98   244.75
                              245.78   250.95   251.42   294.23   297.54
                              306.91   307.17   353.70   370.02   370.32
                              371.89   379.43   384.70   401.04   401.32
                              461.12   480.89   488.98   489.24   637.00
                              665.15   673.70   673.80   732.44   751.09
                              760.87   761.34   761.71   791.61   824.49
                              824.67   843.91   953.05   982.89   988.96
                              989.23   995.04  1039.46  1040.15  1041.82
                             1066.14  1066.64  1215.10  1232.98  1233.28
                             1289.40  1436.87  1437.42  1518.20  1553.55
                             1577.44  2359.08  2359.11  2365.07  2367.94
                             2396.68  2399.88  2399.92  2425.21  2459.96
                             2461.35  2461.48  2496.16  4661.82  4665.26
                             4718.75  4719.83  4858.27  4990.26  4990.61
                             5047.93  5216.46  5221.54  5221.94  5223.23
                             5256.65  5263.12  5263.58  5267.93  5332.12
```

```
                           5333.64  5334.11  5342.74  5576.89  5577.08
                           5577.26  5577.59
 Zero-point correction=                           0.308262 (Hartree/Particle)
 Thermal correction to Energy=                    0.335615
 Thermal correction to Enthalpy=                  0.336560
 Thermal correction to Gibbs Free Energy=         0.254260
 Sum of electronic and zero-point Energies=           -917.390973
 Sum of electronic and thermal Energies=              -917.363619
 Sum of electronic and thermal Enthalpies=            -917.362675
 Sum of electronic and thermal Free Energies=         -917.444975

   0  1
             8         -2.887376   -1.734013   -1.034863
             1         -2.939569   -0.804219   -1.381025
             1         -3.566769   -2.242276   -1.488415
             8         -2.804543    0.872889   -1.712763
             1         -2.935032    1.342788   -0.869352
             1         -1.889967    1.072107   -1.977073
             8         -2.885812    1.733393    1.036614
             1         -2.937688    0.803415    1.382355
             1         -3.563155    2.241996    1.492849
             8         -2.802254   -0.873537    1.713671
             1         -2.932814   -1.343217    0.870096
             1         -1.887877   -1.073168    1.978263
             8          0.054756   -0.861769    1.763770
             1          0.058007    0.100220    1.561214
             1          0.965392   -1.065423    2.042908
             8         -0.053610   -1.763313   -0.862103
             1         -0.059484   -1.559371    0.099651
             1         -0.962762   -2.046173   -1.066767
             8          0.052027    0.862683   -1.763940
             1          0.056107   -0.099119   -1.560289
             1          0.961733    1.066012   -2.046104
             8         -0.053340    1.765199    0.862247
             1         -0.964259    2.044534    1.064280
             1         -0.055018    1.561992   -0.099606
             8          2.887164   -1.036857    1.732713
             1          2.939375   -1.382675    0.802734
             1          3.565337   -1.491758    2.241367
             8          2.803154   -1.714061   -0.873768
             1          1.887899   -1.977228   -1.071515
             1          2.933630   -0.870213   -1.342890
             8          2.884998    1.035861   -1.734290
             1          2.937552    1.381800   -0.804378
             1          3.564161    1.489593   -2.242690
             8          2.804696    1.713645    0.872502
             1          1.891011    1.979230    1.073855
             1          2.935308    0.870192    1.342224

$nbo file=w12_q4_t8 archive nrt plot memory=10gb nrtres=50000 $end

*******************************************************************
%mem=2gb
%nprocshared=8
%chk=w16_q8_t8
#b3lyp/6-311++G** pop=nbo7read

w16_q8_t8 ("3-cube"), E(RB3LYP) = -1223.60719747
nu: 44,45(2),64,...
 Vibrational temperatures:     63.87    64.59    65.27    92.21   100.60
          (Kelvin)            114.32   116.40   116.98   123.36   129.54
                              130.40   143.12   151.59   159.80   159.86
                              162.63   179.98   205.99   230.17   232.50
                              241.82   248.72   256.11   263.91   277.97
                              281.88   286.77   288.09   293.97   305.07
                              310.63   351.71   354.68   356.51   374.32
```

```
                   377.91   379.31   380.43   385.07   387.95
                   402.89   407.21   468.73   474.44   487.54
                   495.10   641.53   659.12   663.56   676.76
                   718.81   731.51   735.93   739.84   742.54
                   756.78   762.72   771.66   783.11   817.05
                   823.79   841.74   877.20   897.78   946.34
                   961.20   972.02   991.37   994.09  1000.31
                  1020.10  1032.79  1044.20  1045.51  1047.98
                  1062.15  1093.57  1193.02  1205.59  1206.44
                  1242.10  1257.71  1305.50  1306.85  1439.48
                  1446.61  1477.24  1534.98  1558.91  1576.76
                  2358.86  2360.10  2365.63  2367.82  2387.30
                  2388.87  2396.03  2412.34  2418.35  2420.09
                  2451.97  2453.55  2461.92  2470.49  2473.36
                  2503.03  4656.24  4657.02  4710.84  4714.56
                  4879.79  4881.88  4982.08  4982.14  5018.53
                  5035.45  5046.40  5076.41  5215.88  5216.45
                  5220.83  5221.21  5248.23  5248.64  5249.02
                  5254.59  5297.20  5301.35  5316.70  5317.44
                  5338.37  5340.61  5340.94  5347.65  5576.24
                  5576.30  5576.63  5576.80
Zero-point correction=                           0.412633 (Hartree/Particle)
Thermal correction to Energy=                    0.449176
Thermal correction to Enthalpy=                  0.450120
Thermal correction to Gibbs Free Energy=         0.347911
Sum of electronic and zero-point Energies=          -1223.194565
Sum of electronic and thermal Energies=             -1223.158021
Sum of electronic and thermal Enthalpies=           -1223.157077
Sum of electronic and thermal Free Energies=        -1223.259287

  0  1
              8         -4.296553   -1.496620    1.356834
              1         -4.353644   -1.556024    0.366439
              1         -4.971037   -2.082308    1.714205
              8         -4.222145   -1.384905   -1.331755
              1         -4.352233   -0.441586   -1.537476
              1         -3.308467   -1.581324   -1.601637
              8         -4.297111    1.496303   -1.356558
              1         -4.354373    1.555716   -0.366168
              1         -4.971867    2.081593   -1.714067
              8         -4.222868    1.384645    1.331974
              1         -4.352523    0.441272    1.537716
              1         -3.309236    1.581442    1.601744
              8         -1.367410    1.437911    1.350343
              1         -1.352839    1.527342    0.371776
              1         -0.480711    1.706230    1.646193
              8         -1.466110   -1.351715    1.438337
              1         -1.455839   -0.373819    1.532210
              1         -2.383022   -1.613979    1.638857
              8         -1.366503   -1.437532   -1.350674
              1         -1.352211   -1.526879   -0.372106
              1         -0.479717   -1.705963   -1.646209
              8         -1.466569    1.352195   -1.438803
              1         -2.383729    1.613692   -1.639185
              1         -1.455525    0.374319   -1.532662
              8          1.465818    1.352323    1.436612
              1          1.455085    0.374547    1.531818
              1          2.383163    1.614133    1.636021
              8          1.367087   -1.437507    1.352170
              1          0.479275   -1.702771    1.647510
              1          1.352674   -1.527852    0.373736
              8          1.466316   -1.353865   -1.436994
              1          1.455089   -0.376076   -1.531728
              1          2.383760   -1.615054   -1.636689
              8          1.367036    1.436347   -1.351579
              1          0.480616    1.703608   -1.649098
```

```
            1         1.350446    1.525641   -0.373004
            8         4.296134    1.499195    1.355131
            1         4.353719    1.557065    0.364654
            1         4.967639    2.088639    1.711905
            8         4.222607    1.383683   -1.333357
            1         3.308292    1.578646   -1.602204
            1         4.353632    0.440402   -1.538414
            8         4.296641   -1.498239   -1.355295
            1         4.354503   -1.556090   -0.364908
            1         4.969082   -2.086526   -1.712251
            8         4.224267   -1.382293    1.333712
            1         3.310640   -1.578630    1.603617
            1         4.354261   -0.438816    1.538622

$nbo file=w16_q8_t8 archive nrt plot memory=10gb nrtres=50000 $end

*****************************************************************
%mem=2gb
%nprocshared=8
%chk=w7_q1_d6
#b3lyp/6-311++G** pop=nbo7read

w7_2_pane (w7_q1_d6), E(RB3LYP) =  -535.304096032
nu: 20,21,35,...
 Vibrational temperatures:     28.89    30.70    50.56    79.55    84.01
          (Kelvin)             96.63   132.97   228.58   245.02   300.16
                              311.94   319.09   322.82   341.51   351.71
                              366.24   372.45   386.20   399.74   411.90
                              437.52   588.58   610.41   639.04   648.12
                              704.38   808.98   954.33  1015.07  1059.35
                             1115.19  1170.67  1216.83  1255.71  1417.00
                             1444.86  2355.29  2363.88  2376.25  2388.92
                             2408.61  2419.36  2432.94  4849.98  4877.72
                             4960.04  4982.73  5021.29  5025.23  5063.16
                             5087.47  5587.36  5587.93  5592.73  5593.48
                             5595.90  5605.01
 Zero-point correction=                           0.174368 (Hartree/Particle)
 Thermal correction to Energy=                    0.192388
 Thermal correction to Enthalpy=                  0.193332
 Thermal correction to Gibbs Free Energy=         0.127129
 Sum of electronic and zero-point Energies=           -535.129728
 Sum of electronic and thermal Energies=              -535.111708
 Sum of electronic and thermal Enthalpies=            -535.110764
 Sum of electronic and thermal Free Energies=         -535.176967

0 1
         8         1.911789   -1.248910   -1.448148
         1         2.730968   -0.874115   -1.053877
         1         2.063596   -2.192978   -1.547023
         8         3.930843    0.002077   -0.079789
         1         3.371962    0.485958    0.568633
         1         4.503985    0.660651   -0.482686
         8         2.046666    1.210961    1.496046
         1         1.224217    0.826568    1.116936
         1         1.962962    1.151094    2.451621
         8         0.006390   -0.030527    0.118645
         1         0.578420   -0.570841   -0.471110
         1        -0.631277   -0.642439    0.546151
         8        -2.074540   -1.538603    1.101115
         1        -2.857052   -1.048888    0.763997
         1        -2.301052   -1.860377    1.977454
         8        -3.936683    0.078122   -0.108849
         1        -3.298534    0.670240   -0.567566
         1        -4.592173    0.652842    0.296011
         8        -1.878703    1.534815   -1.168342
         1        -1.110445    1.062163   -0.776027
```

```
           1         -1.691678    1.616641   -2.107949

$nbo file=w7_q1_d6 archive nrt plot $end

*******************************************************************
%mem=2gb
%nprocshared=8
%chk=w8_q1_t2_d5
#b3lyp/6-311++G** pop=nbo7read

w8_3_pane (w8_q1_t2_d5) (from w2@w6c start), -611.778975556
nu: 23,34,38,...
 Vibrational temperatures:     33.41    49.59    54.46    62.84    89.63
          (Kelvin)             98.15   122.43   136.63   207.89   245.66
                              266.05   282.36   301.27   309.23   317.89
                              328.69   333.90   349.26   376.18   382.50
                              403.70   410.08   446.01   467.49   541.36
                              591.88   624.79   646.07   671.69   816.46
                              832.40   930.74   987.51  1041.06  1090.43
                             1110.15  1167.78  1177.64  1220.82  1249.38
                             1406.41  1574.06  2349.62  2357.04  2364.19
                             2373.97  2402.05  2407.74  2428.16  2507.23
                             4523.50  4768.16  4885.30  4947.49  5015.66
                             5087.37  5142.69  5182.50  5262.52  5275.94
                             5577.48  5584.87  5592.64  5593.70  5594.54
                             5596.56
 Zero-point correction=                           0.200422 (Hartree/Parti$
 Thermal correction to Energy=                    0.220678
 Thermal correction to Enthalpy=                  0.221622
 Thermal correction to Gibbs Free Energy=         0.151112
 Sum of electronic and zero-point Energies=           -611.578553
 Sum of electronic and thermal Energies=              -611.558298
 Sum of electronic and thermal Enthalpies=            -611.557354
 Sum of electronic and thermal Free Energies=         -611.627863

0 1
          8          1.736329    0.965622    1.369414
          1          2.550428    0.577434    0.963085
          1          1.933799    1.122497    2.297875
          8          3.713564   -0.265693    0.005766
          1          3.212105   -0.959323   -0.485992
          1          4.517371   -0.683729    0.326705
          8          2.033750   -2.065766   -1.126656
          1          1.180829   -1.760564   -0.738011
          1          1.880574   -2.160982   -2.071267
          8         -0.106594   -0.913149    0.154090
          1          0.410610   -0.352203    0.759382
          1         -0.558947   -0.257487   -0.449895
          8         -1.366770    0.943616   -1.286463
          1         -2.294772    0.909985   -0.981687
          1         -0.975716    1.775703   -0.963161
          8         -3.853848    0.291825   -0.180135
          1         -3.537569   -0.495155    0.310554
          1         -4.595800   -0.007543   -0.713023
          8         -2.573126   -1.846744    1.044839
          1         -1.637116   -1.638483    0.835273
          1         -2.597876   -2.090790    1.974098
          8          0.268210    2.912680   -0.093139
          1          0.861026    2.331396    0.419032
          1          0.838946    3.510110   -0.584689

$nbo file=w8_q1_t2_d5 archive nrt $end

************************************************************************
%mem=2gb
%nprocshared=8
```

```
%chk=w10_q1_t4_d5
#b3lyp/6-311++G** pop=nbo7read

w10_4_pane (w10_q1_t4_d5), E(RB3LYP) =  -764.725313538
nu: 14,24,30,...
 Vibrational temperatures:     20.51    34.08    43.22    47.38    52.33
          (Kelvin)             71.03    80.41    89.20   119.00   125.94
                              144.27   190.55   211.41   243.38   250.20
                              263.94   284.94   292.90   303.76   307.31
                              313.92   328.61   330.48   356.73   382.67
                              395.25   403.20   418.99   427.08   461.40
                              482.43   535.44   540.33   631.38   654.77
                              673.49   735.09   759.65   819.63   841.53
                              858.09   960.30   991.41  1008.08  1043.13
                             1066.77  1159.51  1183.32  1199.60  1207.70
                             1251.04  1282.16  1415.73  1651.53  2349.11
                             2356.28  2370.50  2371.78  2381.68  2406.14
                             2410.57  2425.53  2471.09  2515.93  4322.20
                             4776.94  4818.58  4910.22  4966.69  4973.54
                             5082.98  5149.20  5186.91  5193.87  5259.35
                             5273.08  5397.59  5576.76  5579.56  5581.21
                             5594.85  5596.18  5597.08  5601.77
 Zero-point correction=                           0.250880 (Hartree/Particle)
 Thermal correction to Energy=                    0.276654
 Thermal correction to Enthalpy=                  0.277598
 Thermal correction to Gibbs Free Energy=         0.192841
 Sum of electronic and zero-point Energies=           -764.474433
 Sum of electronic and thermal Energies=              -764.448660
 Sum of electronic and thermal Enthalpies=            -764.447716
 Sum of electronic and thermal Free Energies=         -764.532473

  0  1
             8          3.150771    0.618464    1.202148
             1          3.763650   -0.001187    0.735041
             1          3.501820    0.746178    2.088833
             8          4.497432   -1.168370   -0.308007
             1          3.753461   -1.657903   -0.732651
             1          5.157846   -1.823917   -0.067316
             8          2.215460   -2.302714   -1.246407
             1          1.557204   -1.774071   -0.738595
             1          1.953088   -2.239339   -2.169693
             8          0.696993   -0.617300    0.319313
             1          1.438373   -0.234282    0.823029
             1          0.378056    0.143499   -0.258433
             8         -0.144492    1.456287   -1.063732
             1         -0.999676    1.698569   -0.642356
             1          0.500910    2.156284   -0.859721
             8         -2.486173    1.665999    0.355398
             1         -2.338889    0.929768    0.967874
             1         -3.320661    1.438063   -0.098520
             8         -1.690436   -0.943842    1.596774
             1         -0.777854   -0.911112    1.218034
             1         -1.600958   -1.170159    2.527422
             8          2.146322    2.887482   -0.209372
             1          2.608436    2.170622    0.262610
             1          2.802701    3.303904   -0.775028
             8         -4.795468    0.524901   -0.806370
             1         -4.586168   -0.405157   -0.587871
             1         -4.992693    0.543226   -1.746850
             8         -3.853287   -1.931097    0.127359
             1         -3.058965   -1.679418    0.639979
             1         -3.636658   -2.752050   -0.322618

$nbo file=w10_q1_t4_d5 archive nrt plot $end

*******************************************************************
```

```
%mem=2gb
%nprocshared=8
%chk=w12_q3_t2_d7
#b3lyp/6-311++G** pop=nbo7read

w12_5_pane (w12_q3_t2_d7), E(RB3LYP) =  -917.678777032
nu: 9,19,25,...
 Vibrational temperatures:     13.33    27.82    36.39    44.29    47.12
          (Kelvin)             53.85    61.36    77.69    80.69    89.58
                              109.06   114.91   128.51   134.66   175.72
                              194.72   221.67   245.29   263.00   271.08
                              279.47   295.59   304.63   309.68   324.54
                              330.87   332.66   336.85   346.06   354.28
                              367.77   376.55   399.38   401.78   408.24
                              429.46   445.23   484.11   544.95   584.86
                              603.34   640.40   667.11   704.19   769.75
                              787.25   817.59   838.45   890.41   934.04
                              955.54  1005.11  1028.31  1056.13  1108.64
                             1121.03  1140.10  1159.56  1185.41  1197.25
                             1212.95  1239.63  1283.00  1416.14  1456.15
                             1583.68  2352.91  2358.33  2371.33  2377.11
                             2384.96  2396.03  2400.90  2405.29  2423.22
                             2437.09  2482.06  2504.38  4622.48  4789.29
                             4876.37  4899.69  4953.38  4970.10  4990.08
                             5014.19  5060.29  5081.22  5117.83  5133.96
                             5156.47  5193.88  5235.73  5266.32  5574.08
                             5586.10  5590.50  5592.52  5593.48  5597.15
                             5600.62  5612.38
 Zero-point correction=                           0.302194 (Hartree/Particle)
 Thermal correction to Energy=                    0.333166
 Thermal correction to Enthalpy=                  0.334110
 Thermal correction to Gibbs Free Energy=         0.236318
 Sum of electronic and zero-point Energies=           -917.376583
 Sum of electronic and thermal Energies=              -917.345611
 Sum of electronic and thermal Enthalpies=            -917.344667
 Sum of electronic and thermal Free Energies=         -917.442459

   0  1
              8         -3.075179   -1.265748    0.622555
              1         -3.893211   -0.720308    0.721684
              1         -3.027105   -1.853565    1.383043
              8         -5.083186    0.541513    0.661123
              1         -4.587503    1.362208    0.428911
              1         -5.691692    0.779223    1.366206
              8         -3.402097    2.589294    0.055451
              1         -2.542763    2.106599    0.035095
              1         -3.443189    3.101830   -0.757120
              8         -1.246828    0.889642    0.117494
              1         -1.756721    0.118236    0.429248
              1         -0.916778    0.605188   -0.778069
              8         -0.327931   -0.136649   -2.194657
              1          0.581179   -0.434800   -2.003870
              1         -0.881176   -0.934906   -2.283062
              8          2.227839   -0.612006   -1.039798
              1          1.930608   -0.070145   -0.277342
              1          3.004230   -0.123895   -1.386744
              8          1.319663    0.824380    1.206153
              1          0.372891    0.942730    0.978663
              1          1.374279    0.115289    1.883087
              8         -2.242518   -2.188265   -1.930427
              1         -2.632843   -1.956550   -1.066964
              1         -2.980109   -2.374485   -2.518097
              8          4.337803    1.125249   -1.581749
              1          4.151857    1.720608   -0.826029
              1          4.354196    1.684742   -2.363447
              8          3.455755    2.462992    0.675817
```

```
              1          2.645954    1.989104    0.963306
              1          3.388209    3.362802    1.004387
              8          1.598628   -1.441297    2.750294
              1          1.967661   -2.048903    2.071563
              1          2.169231   -1.516380    3.520204
              8          2.514284   -2.833589    0.558785
              1          2.500504   -2.137787   -0.136919
              1          3.302423   -3.360946    0.403933

$nbo file=w12_q3_t2_d7 archive nrt $end

*****************************************************************
%mem=2gb
%nprocshared=8
%chk=w14_q3_t8_d3
#b3lyp/6-311++G** pop=nbo7read

w14_6p (w14_q3_t8_d3), E(RB3LYP) =  -1070.63164768
nu: 9,15,27,....
 Vibrational temperatures:     13.61    21.20    39.21    48.96    54.32
          (Kelvin)             71.89    77.74    88.89   102.06   114.28
                              116.13   124.70   130.26   136.00   160.42
                              180.54   197.07   205.01   230.08   239.29
                              244.87   256.65   267.41   277.17   289.13
                              297.48   303.41   305.23   322.23   342.32
                              349.50   361.15   368.96   390.06   393.04
                              400.81   411.05   424.09   438.55   448.69
                              457.43   501.57   510.46   611.78   628.01
                              634.42   657.96   683.12   697.04   714.87
                              728.88   765.24   784.24   825.09   830.82
                              859.56   874.04   880.10   913.47   946.16
                              956.93   984.84  1001.97  1032.13  1054.82
                             1067.23  1095.21  1136.82  1145.88  1165.68
                             1171.78  1201.93  1231.67  1302.38  1398.32
                             1438.98  1507.38  1657.74  2347.83  2361.36
                             2364.22  2375.92  2376.24  2393.38  2398.53
                             2412.91  2427.33  2440.94  2454.39  2474.55
                             2483.01  2511.33  4351.35  4718.55  4758.49
                             4891.84  4938.42  4965.03  4995.16  5010.39
                             5027.14  5096.99  5150.94  5157.19  5173.51
                             5254.94  5262.76  5272.78  5291.82  5337.03
                             5342.83  5382.68  5388.58  5559.61  5573.77
                             5574.16  5578.46  5584.00  5592.74  5595.44
 Zero-point correction=                           0.356184 (Hartree/Particle)
 Thermal correction to Energy=                    0.390613
 Thermal correction to Enthalpy=                  0.391557
 Thermal correction to Gibbs Free Energy=         0.288126
 Sum of electronic and zero-point Energies=          -1070.275464
 Sum of electronic and thermal Energies=             -1070.241034
 Sum of electronic and thermal Enthalpies=           -1070.240090
 Sum of electronic and thermal Free Energies=        -1070.343522

   0  1
              8         -4.376155   -1.658535   -0.769855
              1         -3.908854   -1.988313    0.016363
              1         -4.772469   -0.818721   -0.478702
              8         -2.626073   -1.992290    1.507595
              1         -2.733221   -1.115370    1.934573
              1         -2.589576   -2.647464    2.211787
              8         -2.897795    0.704252    2.160522
              1         -2.142377    1.045399    1.590457
              1         -2.802761    1.117862    3.024925
              8         -2.421644   -0.978518   -2.491401
              1         -3.201434   -1.316868   -1.981977
              1         -2.452758   -1.381261   -3.364856
              8         -0.558634   -1.244679   -0.373328
```

```
               1         -1.140083   -1.709925    0.252799
               1         -1.029767   -1.262273   -1.228858
               8         -1.026777    1.397476    0.444488
               1         -0.727394    0.509482    0.151061
               1         -1.549191    1.727402   -0.323526
               8          2.172432   -0.681883    0.252529
               1          1.336053   -1.087365   -0.029021
               1          2.751757   -1.388824    0.614292
               8          1.731065    1.903244    1.317517
               1          0.806941    2.020251    1.048136
               1          1.926864    0.974696    1.082571
               8          4.216823    0.584195   -1.324876
               1          3.404662    0.190920   -0.958411
               1          4.224347    1.496830   -0.974515
               8          3.834372    3.066656   -0.043313
               1          3.052297    2.797949    0.488515
               1          3.586540    3.879865   -0.491409
               8          4.088264   -2.477994    1.074668
               1          4.835348   -2.154361    0.517118
               1          4.414666   -2.460667    1.978999
               8          5.962185   -1.289627   -0.499175
               1          5.430426   -0.526574   -0.842722
               1          6.387409   -1.686427   -1.264300
               8         -2.781334    1.783497   -1.640935
               1         -3.631914    1.656787   -1.191922
               1         -2.643701    0.965813   -2.149708
               8         -4.910548    0.978639    0.242086
               1         -4.347198    0.985375    1.044077
               1         -5.734053    1.420323    0.472078

$nbo file=w14_q3_t8_d3 archive $end

****************************************************************
%mem=2gb
%nprocshared=8
%chk=w14_q4_t4_d6
#b3lyp/6-311++G** pop=nbo7read

w14_6_pane (w14_q4_t4_d6), E(RB3LYP) =  -1070.63220856
nu: 16,18,29,...
 Vibrational temperatures:     22.96    26.34    41.39    48.62    53.58
          (Kelvin)             59.27    60.92    71.49    78.82    90.96
                              100.06   106.47   111.36   128.62   134.54
                              136.03   182.71   195.81   214.87   222.45
                              229.05   240.59   260.36   269.78   278.77
                              286.84   293.30   304.78   306.67   313.05
                              318.32   322.74   332.94   341.77   349.92
                              361.82   377.00   384.32   402.31   412.36
                              427.03   434.73   444.43   471.27   592.30
                              594.85   596.00   620.87   701.59   730.00
                              735.60   760.40   777.03   792.25   829.41
                              888.82   904.22   911.60   915.08   929.61
                              998.10  1008.28  1029.39  1037.99  1102.64
                             1112.15  1118.27  1151.44  1163.07  1187.16
                             1209.45  1251.20  1265.96  1299.00  1416.44
                             1438.84  1501.23  1546.31  2353.09  2361.88
                             2363.96  2372.78  2384.44  2391.76  2392.96
                             2397.40  2403.41  2422.34  2423.74  2463.48
                             2480.69  2488.27  4701.40  4772.62  4823.88
                             4870.39  4887.48  4938.16  4941.86  5002.40
                             5036.61  5049.08  5083.45  5094.75  5111.34
                             5144.86  5170.32  5184.26  5212.33  5233.05
                             5309.68  5320.92  5579.74  5580.19  5595.00
                             5598.54  5598.72  5601.48  5602.82  5609.01
 Zero-point correction=                           0.354244 (Hartree/Particle)
 Thermal correction to Energy=                    0.389778
```

```
 Thermal correction to Enthalpy=                 0.390723
 Thermal correction to Gibbs Free Energy=        0.284144
 Sum of electronic and zero-point Energies=          -1070.277965
 Sum of electronic and thermal Energies=             -1070.242430
 Sum of electronic and thermal Enthalpies=           -1070.241486
 Sum of electronic and thermal Free Energies=        -1070.348064

   0  1
              8          2.416491   -2.434217   -0.151069
              1          3.206778   -2.186202   -0.691190
              1          2.420495   -3.392673   -0.068481
              8          4.378606   -1.345355   -1.647472
              1          3.936484   -0.495442   -1.891180
              1          4.838825   -1.652068   -2.433233
              8          2.833601    0.819231   -2.182533
              1          1.962804    0.387494   -2.099444
              1          2.829748    1.503418   -1.491561
              8          0.508695   -0.638253   -1.288117
              1          1.021892   -1.412561   -0.987963
              1          0.470562   -0.058475   -0.494114
              8          0.445683    0.953246    0.988991
              1         -0.499272    0.981372    1.246232
              1          0.941650    0.338295    1.588498
              8         -2.323306    0.729192    1.113925
              1         -2.309012    0.248590    0.255877
              1         -2.905221    1.499632    0.942713
              8         -2.265581   -0.731207   -1.275004
              1         -1.305909   -0.844558   -1.444829
              1         -2.623915   -1.593636   -0.969085
              8          2.182360   -0.703464    2.185652
              1          2.355459   -1.379122    1.507664
              1          2.983040   -0.146766    2.221780
              8         -4.072143    2.641571    0.115232
              1         -4.128095    2.264648   -0.788490
              1         -3.951434    3.588871    0.007700
              8         -3.964733    1.195249   -2.232939
              1         -3.341700    0.461042   -2.040549
              1         -3.904063    1.374937   -3.174504
              8         -3.314153   -2.924122   -0.001718
              1         -3.421884   -2.551329    0.900018
              1         -4.148153   -3.347432   -0.223336
              8         -3.367699   -1.511898    2.368287
              1         -3.041454   -0.629712    2.086579
              1         -4.036409   -1.358121    3.040846
              8          4.182255    1.264794    1.939789
              1          3.717512    1.870312    1.332755
              1          4.586026    1.812135    2.618798
              8          2.424749    2.569482    0.135429
              1          1.599227    2.137863    0.467308
              1          2.227442    3.505503    0.033559

$nbo file=w14_q4_t4_d6 archive nrt plot $end

****************************************************************
%mem=4GB
 %nprocshared=8
 %chk=w16_q8_d8
 #N B3LYP/6-311++G** POP=NBO6Read

 w16_7_pane (w16_q8_d8), E(RB3LYP) =  -1223.5921758
 nu: 20,25,29,...
 Vibrational temperatures:     28.19    35.44    42.25    44.58    47.35
          (Kelvin)             50.72    55.81    60.90    61.91    92.05
                               97.10   102.51   111.57   114.14   118.62
                              135.05   148.16   154.97   164.71   186.55
                              198.38   209.43   217.59   247.40   253.21
```

```
                    270.42   271.56   280.63   282.58   288.97
                    290.76   303.93   307.12   311.47   313.66
                    338.77   340.87   345.09   356.63   361.21
                    372.78   375.25   378.27   383.11   392.72
                    401.57   418.66   435.74   445.53   458.01
                    593.48   615.70   619.83   622.04   635.03
                    637.36   652.05   768.09   776.27   800.14
                    826.39   845.03   883.26   892.22   898.21
                    930.15   952.36   991.87  1002.00  1031.51
                   1038.29  1062.07  1080.48  1092.31  1102.61
                   1112.14  1135.85  1147.48  1168.05  1209.38
                   1221.96  1230.76  1240.96  1252.51  1291.87
                   1352.67  1379.42  1466.12  1487.82  1501.52
                   2353.75  2358.74  2359.43  2363.81  2381.82
                   2384.42  2386.39  2393.79  2400.12  2403.48
                   2425.88  2463.31  2472.89  2473.30  2487.12
                   2500.89  4846.66  4882.88  4926.88  4970.69
                   4977.96  4983.97  4985.47  5005.45  5018.38
                   5028.30  5034.54  5062.80  5079.44  5081.96
                   5098.55  5103.56  5111.67  5127.09  5150.87
                   5153.52  5171.01  5185.24  5190.45  5208.68
                   5588.57  5591.07  5591.38  5596.84  5597.67
                   5598.74  5601.38  5606.55
Zero-point correction=                          0.406965 (Hartree/Particle)
Thermal correction to Energy=                   0.446898
Thermal correction to Enthalpy=                 0.447842
Thermal correction to Gibbs Free Energy=        0.332048
Sum of electronic and zero-point Energies=         -1223.185211
Sum of electronic and thermal Energies=            -1223.145278
Sum of electronic and thermal Enthalpies=          -1223.144334
Sum of electronic and thermal Free Energies=       -1223.260128

  0  1
             8        -1.380573   -1.959443   -0.092851
             1        -1.927699   -2.681500    0.284925
             1        -0.445719   -2.247670   -0.114047
             8        -3.066345   -3.557316    1.369066
             1        -3.227002   -2.946263    2.121017
             1        -2.932670   -4.429768    1.749451
             8        -3.270915   -1.597528    3.305975
             1        -2.660887   -0.911281    2.960528
             1        -4.094773   -1.146158    3.511204
             8        -1.410777   -0.070526    1.928136
             1        -1.433249   -0.724967    1.191050
             1        -1.536977    0.790277    1.481694
             8        -1.347454    2.073678    0.044910
             1        -0.403359    2.326123    0.094299
             1        -1.373418    1.398228   -0.670651
             8         1.456824    2.010462    0.092332
             1         1.484168    1.302456    0.770673
             1         2.155184    2.638358    0.374548
             8         1.409492   -0.119501    1.945933
             1         0.456729   -0.125480    2.174869
             1         1.535141   -0.883155    1.343089
             8        -1.458804    0.122519   -1.926979
             1        -1.660333   -0.710386   -1.456000
             1        -2.244307    0.396597   -2.447271
             8         3.512182    3.361492    1.373638
             1         3.591423    2.702999    2.096510
             1         3.455240    4.220837    1.800839
             8         3.380523    1.325929    3.237248
             1         2.701912    0.706802    2.895292
             1         4.086282    0.784695    3.601441
             8         1.400547   -1.938380   -0.165651
             1         1.414060   -1.229347   -0.844094
             1         2.066866   -2.584170   -0.483504
```

```
              8          1.317283    0.185215   -2.015936
              1          1.497021    0.954171   -1.434819
              1          0.356532    0.212449   -2.204150
              8         -3.659046    1.342174   -2.995638
              1         -3.709232    2.145925   -2.435850
              1         -3.911228    1.602173   -3.885391
              8         -3.473184    3.385464   -1.132581
              1         -2.693293    3.098422   -0.610221
              1         -3.416182    4.341429   -1.210889
              8          3.320858   -3.368334   -1.560275
              1          3.376914   -2.716774   -2.291863
              1          4.226182   -3.540274   -1.286297
              8          3.223542   -1.289522   -3.374715
              1          2.559575   -0.665196   -3.014064
              1          3.073871   -1.330626   -4.323224

 $NBO file=w16_q8_d8 archive nrt memory=10gb nrtres=30000 plot $END

*********************************************************************
%mem=10gb
%nprocshared=20
%chk=w16_q4_t12
#b3lyp/6-311++G** pop=nbo7read

w16_q4_t12 ("dicubane"), E(RB3LYP) =  -1223.58854441
nu: 9,20,33,...
 Vibrational temperatures:     12.23    28.51    47.91    58.20    69.38
          (Kelvin)             72.59   109.32   110.58   111.22   115.93
                              120.46   123.87   131.84   136.89   141.98
                              165.78   174.93   192.54   205.37   219.40
                              222.19   235.58   238.08   241.53   251.27
                              261.48   270.63   284.34   292.49   295.86
                              300.59   312.55   319.11   320.35   347.35
                              358.58   377.53   383.36   392.84   401.53
                              410.79   435.91   437.03   452.14   457.23
                              497.09   506.01   508.04   626.33   632.24
                              655.07   679.72   687.10   702.82   722.06
                              733.57   758.61   770.43   782.09   809.05
                              825.92   842.35   862.26   874.01   876.49
                              907.18   918.83   935.62   958.01   983.36
                              996.38  1006.43  1020.02  1035.89  1055.04
                             1063.23  1084.41  1108.74  1148.17  1149.70
                             1171.59  1175.58  1215.90  1287.59  1321.22
                             1406.65  1439.35  1440.74  1645.64  1652.47
                             2346.56  2360.89  2361.67  2375.68  2378.08
                             2396.20  2401.46  2425.88  2429.06  2440.01
                             2440.84  2452.41  2473.55  2484.00  2492.71
                             2509.69  4349.06  4392.36  4753.72  4756.39
                             4907.78  4912.42  4963.07  5014.26  5032.15
                             5085.50  5105.06  5123.51  5147.33  5156.90
                             5256.05  5268.26  5273.59  5276.05  5282.65
                             5291.61  5322.19  5342.71  5344.90  5369.64
                             5388.98  5392.32  5558.24  5572.02  5573.09
                             5577.31  5579.14  5585.51
 Zero-point correction=                           0.409866 (Hartree/Particle)
 Thermal correction to Energy=                    0.447916
 Thermal correction to Enthalpy=                  0.448860
 Thermal correction to Gibbs Free Energy=         0.339527
 Sum of electronic and zero-point Energies=          -1223.178679
 Sum of electronic and thermal Energies=             -1223.140628
 Sum of electronic and thermal Enthalpies=           -1223.139684
 Sum of electronic and thermal Free Energies=        -1223.249017

   0  1
              8          5.312777    0.993429    0.891361
              1          4.747172    0.646344    1.601650
```

```
1          5.546172    0.208897    0.364885
8          3.206968   -0.259911    2.438355
1          3.130629   -1.109850    1.954029
1          3.143397   -0.463567    3.376995
8          3.030001   -2.333862    0.581418
1          2.359743   -1.899148   -0.027311
1          2.730383   -3.237523    0.726622
8          3.648228    2.441736   -0.647746
1          4.357959    2.045360   -0.080851
1          3.888663    3.359172   -0.811410
8          1.468921    1.321412    0.749913
1          1.965455    0.939207    1.494330
1          2.104327    1.900753    0.283965
8          1.441585   -0.907226   -0.970384
1          1.326711   -0.117413   -0.398878
1          2.063551   -0.592613   -1.669049
8         -1.376542    1.328838    0.489138
1         -0.450846    1.510980    0.723839
1         -1.850651    1.072952    1.305631
8         -1.460810   -0.988811   -1.052161
1         -0.535881   -1.190416   -1.267950
1         -1.416279   -0.134545   -0.568365
8         -3.746691    2.287297   -1.000944
1         -2.855227    2.230637   -0.624301
1         -3.777630    1.566553   -1.664052
8         -3.830162   -0.122640   -2.476220
1         -2.981321   -0.531388   -2.237244
1         -4.488559   -0.618371   -1.961271
8         -3.144218    0.292959    2.392111
1         -3.983202    0.614016    1.939032
1         -3.256511    0.463368    3.331997
8         -5.226796    1.065476    0.985791
1         -4.817802    1.622846    0.282502
1         -5.542654    0.272545    0.518382
8          3.501760    0.149044   -2.441533
1          4.265510   -0.329704   -2.082742
1          3.566520    1.042989   -2.063885
8          5.327260   -1.391841   -0.711685
1          4.627548   -1.916809   -0.270588
1          6.033051   -2.002610   -0.946447
8         -5.431536   -1.446527   -0.416450
1         -4.667305   -1.847853    0.070467
1         -6.108568   -2.125510   -0.489285
8         -3.165272   -2.173323    0.868538
1         -2.485161   -1.945918    0.204469
1         -3.082981   -1.481771    1.548828

$nbo file=w16_q4_t12 archive nrt plot memory=30gb nrtres=50000 $end

********************************************************************
```
